# InterCells: A Generic Monte-Carlo Simulation of Intercellular Interfaces Captures Nanoscale Patterning at the Immune Synapse

**DOI:** 10.3389/fimmu.2018.02051

**Published:** 2018-09-11

**Authors:** Yair Neve-Oz, Julia Sajman, Yair Razvag, Eilon Sherman

**Affiliations:** Racah Institute of Physics, The Hebrew University, Jerusalem, Israel

**Keywords:** cell signaling, T cell activation, kinetic segregation model, single molecule localization microscopy, photoactivated localization microscopy, direct STORM, microvilli, agent based Monte-Carlo simulation

## Abstract

Molecular interactions across intercellular interfaces serve to convey information between cells and to trigger appropriate cell functions. Examples include cell development and growth in tissues, neuronal and immune synapses (ISs). Here, we introduce an agent-based Monte-Carlo simulation of user-defined cellular interfaces. The simulation allows for membrane molecules, embedded at intercellular contacts, to diffuse and interact, while capturing the topography and energetics of the plasma membranes of the interface. We provide a detailed example related to pattern formation in the early IS. Using simulation predictions and three-color single molecule localization microscopy (SMLM), we detected the intricate mutual patterning of T cell antigen receptors (TCRs), integrins and glycoproteins in early T cell contacts with stimulating coverslips. The simulation further captures the dynamics of the patterning under the experimental conditions and at the IS with antigen presenting cells (APCs). Thus, we provide a generic tool for simulating realistic cell-cell interfaces, which can be used for critical hypothesis testing and experimental design in an iterative manner.

## Introduction

Cells associate and form functional interfaces to create tissues, to exchange molecular content and to convey information. Such interfaces form in multicellular organisms between adherent and developing cells in tissues (1), between neurons (2) and immune cells (3). Cell contacts can also occur in unicellular organisms, e.g., between bacteria in biofilms and between bacteria and their host cells (4).

A wide range of physical structures appear in intercellular interfaces, including junctions (e.g., plasmodesmata and gap junctions, tight junctions, and desmosomes) (5), neuronal synapses and immune synapses (IS). The dynamics of the interfaces may vary widely, from seconds to days. For instance, neuronal synapses may persist over much longer times, but still show surprising remodeling dynamics (6, 7).

**Figure 1:**
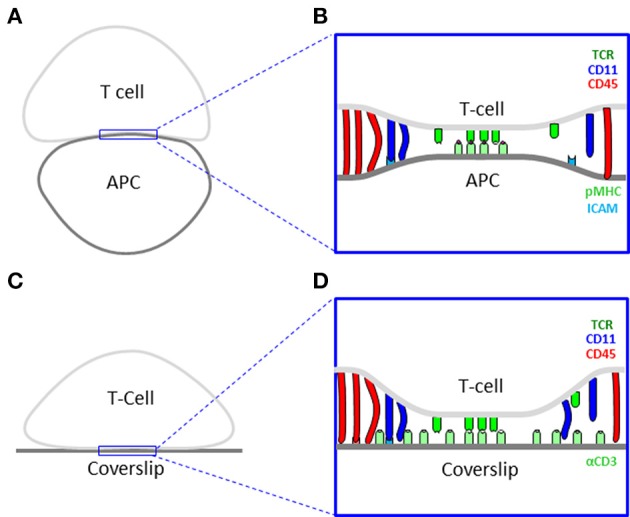
Cell interfaces of the immune synapse. **(A)** A scheme of a physiological interface between a T cell and an antigen presenting cell (APC). **(B)** A physical model of the interface in panel **(A)**. **(C)** A scheme of an experimental interface between a T cell and a coverslip. **(D)** A physical model of the interface in panel **(C)**.

In this study, we focus on the IS between CD4^+^ T Cells and antigen presenting cells (APCs) as an example of a dynamic intercellular interface of outstanding importance and interest (Figures 1A,B). This synapse serves T cells to probe the outer surface of APCs for cognate antigens, and to mount an appropriate immune response (8). Advancements in microscopy have shown that such structures demonstrate complex levels of dynamic organization (9). The IS starts with early contacts that mature within a few minutes to form molecular segregation into supramolecular activating clusters (SMACs) (10). Such experiments often turn to artificial mimics of the APC for high resolution microscopy. Examples include coverslips coated with antibodies (11) (Figures 1C,D) or with lipid bilayers that include molecules of interest (12).

Recently, super-resolution cell imaging, and especially single molecule localization microscopy, has allowed to resolve the organization of molecules in live cells with resolution down to ~20 nm (13). Such methods include Photoactivated Localization Microscopy (PALM) (14) and direct Stochastic Optical Reconstruction Microscopy (dSTORM) (15). Through these techniques, whole (or a large part of) molecular populations of specific protein species can be directly visualized with such resolution. For instance, imaging of signaling molecules in CD4^+^ T cells has shown surprising nanoscale patterning of proteins, in the form of hierarchical and functional clusters (16, 17). Specifically, the nanoscale segregation of the TCRs from bulky glycoproteins, such as CD45, has been detected (18). Still, the latter patterns of kinetic segregation in early contacts (18–20) have not been related to the hierarchical ordering of TCRs, integrins and glycoproteins into central, proximal and distal SMACs (c-, p- and d-SMACs; known also as the “bull's eye” pattern) that has been detected at the mature IS (10, 21, 22).

For gaining further insight on the structure, dynamics and functional role of interfaces, experimental techniques can be complemented with computational cell modeling and simulations. Indeed, multiple computational simulations have been developed and employed for studying cells (23). Such methods may vary widely in their details, from atoms to entire cells, time-scale, from microseconds to minutes and length-scales, from angstroms to microns and more.

Here, we introduce an agent-based Monte-Carlo simulation of user-defined cellular interfaces. The simulation, called InterCells, is based on detailed physical modeling of the interface and embedded molecules within. The simulation allows for the molecules to diffuse and interact, while capturing the topography and energetics of the interacting plasma membranes (PMs). It relies on simple and inexpensive computation that is still complex enough to capture realistic complexity and dynamics of the interfaces. Recently, similar modeling and simulations have served to resolve possible mechanisms of cooperativity and localized activation in TCR clusters (24) and to identify kinetic segregation of TCR and glycoproteins at the engaged tips of microvilli (18).

A special emphasis in our simulation is its easy operation by non-experts. For that, we provide a friendly graphical user interface (GUI) for rapid configuration and deployment of the simulation. Multiple analytical tools are provided for data analysis and interpretation. A key property of the simulation is its ability to confront the results and predictions of realistic simulations with experimental data, acquired by single molecule localization microscopy. We provide a detailed example related to pattern formation in intercellular contacts that characterize the early IS between CD4^+^ T cells and antigen presenting cells (APCs) (18, 20). Specifically, our simulation results predict a new feature of pattern formation—the intricate mutual patterning of T cell antigen receptors (TCRs), integrins and glycoproteins in the early contacts. We confirm this patterning by SMLM imaging of T cells on functionally-coated coverslips. Thus, we provide a generic tool for simulating cell-cell interfaces, which can be used for critical hypothesis testing and experimental design in an iterative manner.

## Results

### Intricate patterning of membrane proteins at the IS

**Figure 2:**
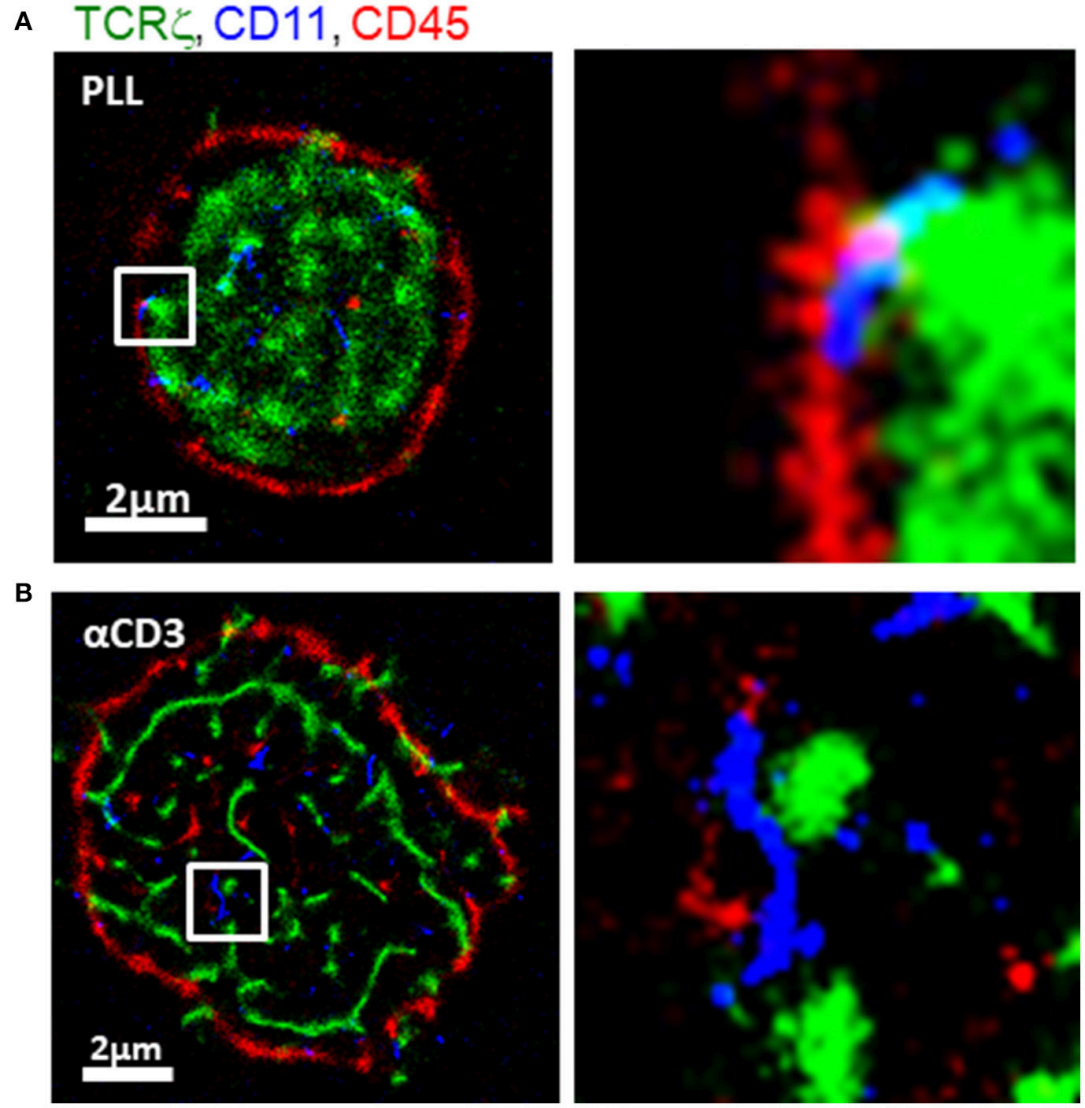
Intricate molecular patterning at the immune synapse. Three-color PALM/dSTORM imaging of fixed E6.1 Jurkat cells expressing TCRζ-Dronpa (green) and stained for CD45 (red) and CD11 (blue). The cells were dropped on coverslips coated with either **(A)** poly-L-lysine (PLL; top raw) or **(B)** αCD3 (bottom raw). Cells were let spread on the coverslip for 1.5 min before fixation. Shown are representative cells (*N* = 40 cells on PLL and 31 cells on αCD3). Bars−2 μm.

To study molecular patterning at the IS, we imaged Jurakt E6.1 CD4^+^ T cells, as they adhered and spread on functionally coated coverslips (11) (see details in Materials and Methods). Anti-CD3-coated coverslips result in direct TCR stimulation, T cell activation and spreading. In contrast, coverslips coated with poly-L-lysine (PLL) show reduced levels of TCR stimulation and smaller cell footprints (25). For imaging, we used three-color single molecule localization microscopy (SMLM) in total internal reflection (TIRF; Figure 2). Our SMLM approach included PALM imaging of TCRζ-Dronpa, stably expressed by the cells. CD11 and CD45 molecules were immunostained using an anti-CD11-Alexa568 and anti-CD45-Alexa647, respectively (see Materials and Methods) and imaged by two-color dSTORM.

Our images showed a striking patterning of molecules where CD11 clusters (blue) localized in between TCR clusters (green) and CD45 clusters (red) on either TCR-stimulating and non-stimulating conditions. On PLL-coated coverslips, TCR clusters occupied the center of the interface, while CD45 showed an outer ring. CD11 clusters localized outside TCR clusters and in the formed gaps between TCRs and CD45. On αCD3-coated coverslips, CD11 also localized in between TCR and CD45. However, on these coverslips, the cells formed larger footprints and TCR was more clustered.

The segregation of TCRs from CD45 has been shown by diffraction limited microscopy, and more recently, in early contact (18). Also, the localization of CD11 was shown before in the pSMAC while CD45 localized to the dSMAC (22). However, such mutual patterning has not been resolved in early ISs and at the nanoscale. Thus, our imaging captured an intricate mutual patterning of TCRs, integrins and glycoproteins in the early contacts. The occurrence of the mutual patterning on coverslips coated with either αCD3 or PLL indicates that this patterning is caused by the physical contact of the cell with the opposing interface of the coverslip.

### Modeling and simulation of molecular patterning under the experimental conditions

**Figure 3:**
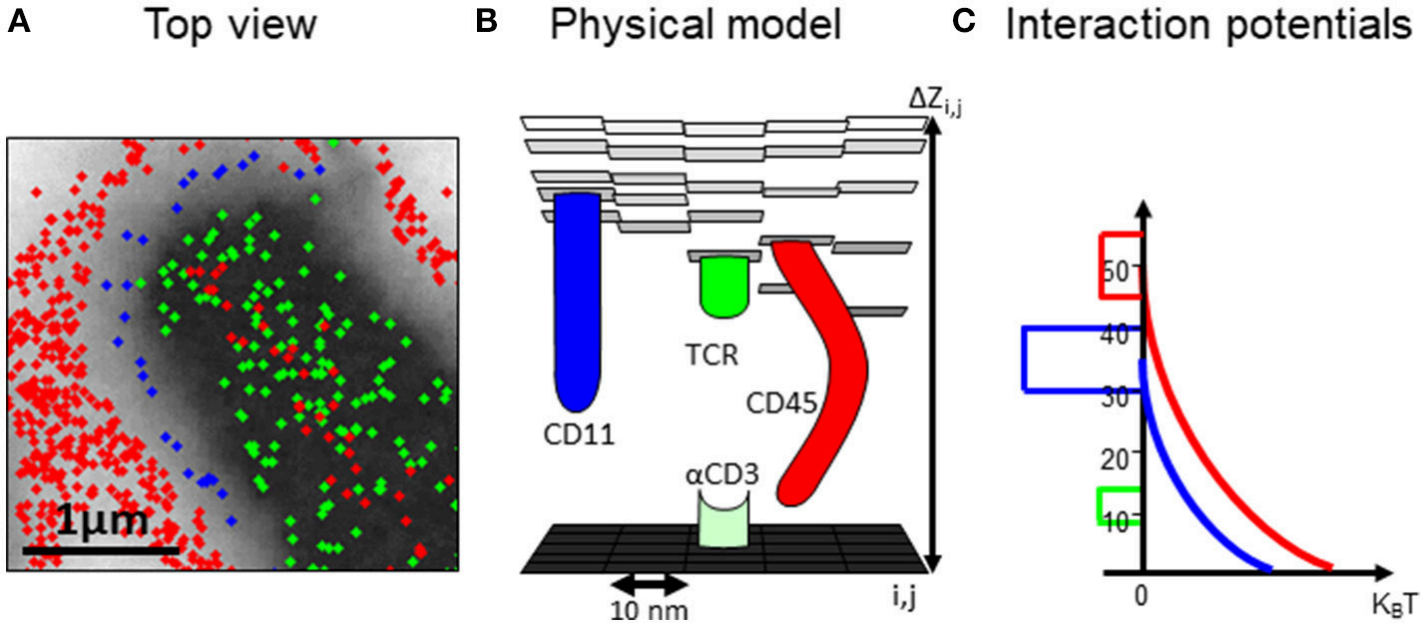
A computational model of the intercellular interface between T cells and a coverslip. **(A)** A top view of a simulated membrane. The membrane is shown as a grid, where molecules (colored circles) are embedded and can diffuse and interact. Membrane height is marked by variable gray levels. **(B)** A 3D view of the simulated membrane. **(C)** The interaction potential between molecules embedded in the simulated membrane.

**Figure 4:**
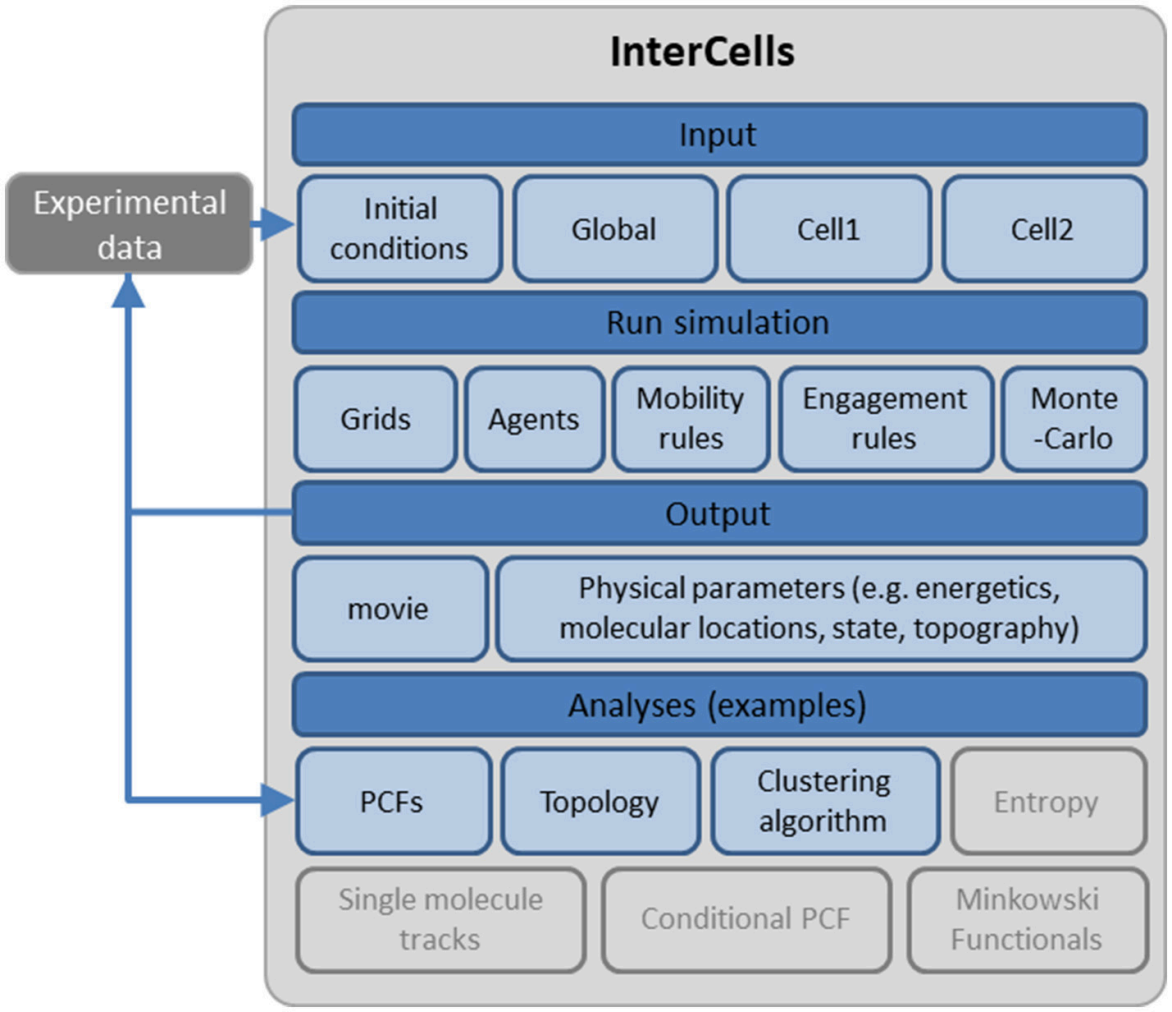
A schematic description of the cell interface simulation. A detailed account of the simulation, its structure and interface are provided as a User's Guide in the Supplemental Information.

**Figure 5:**
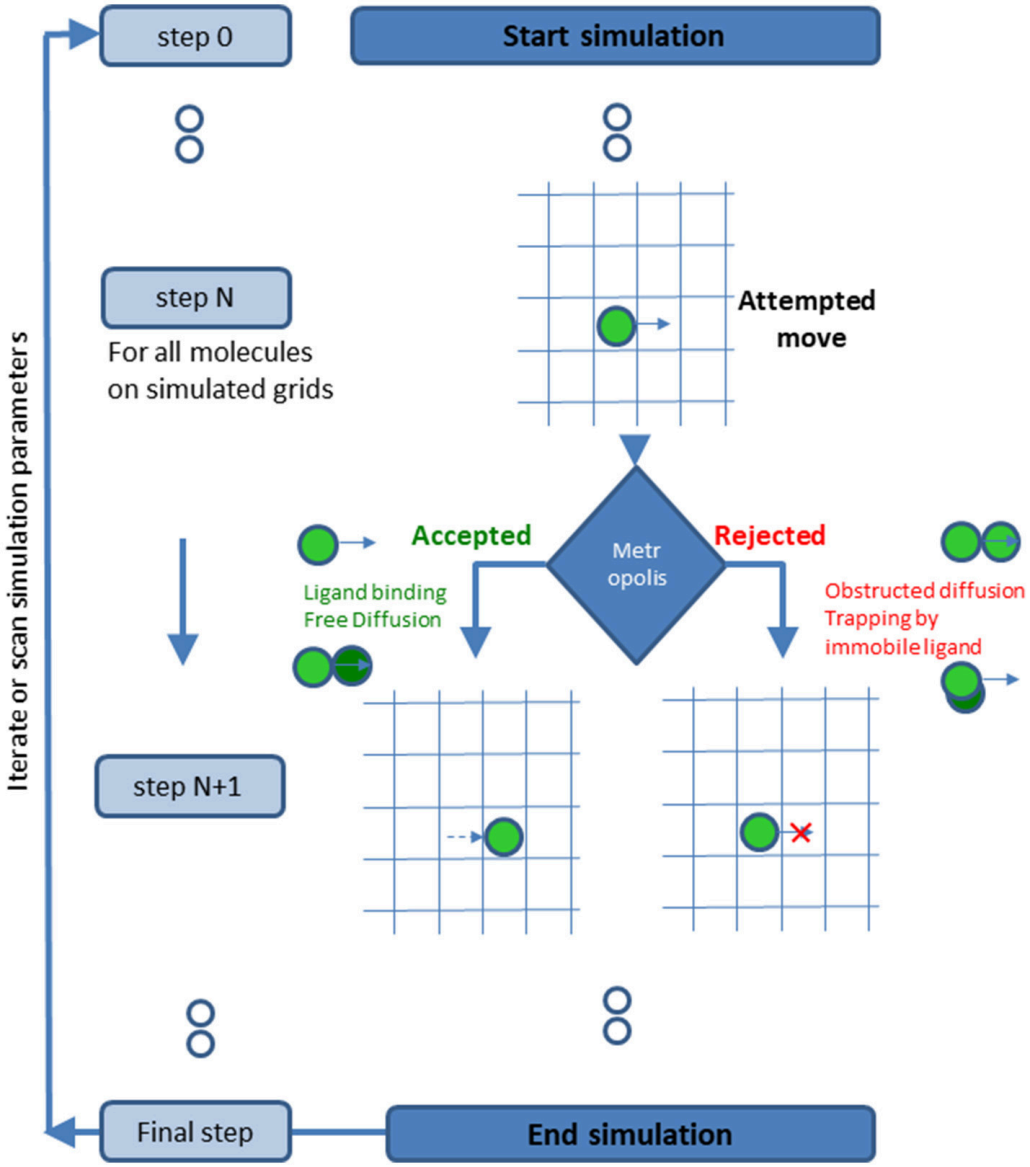
A schematic description of the simulation process.

**Figure 6:**
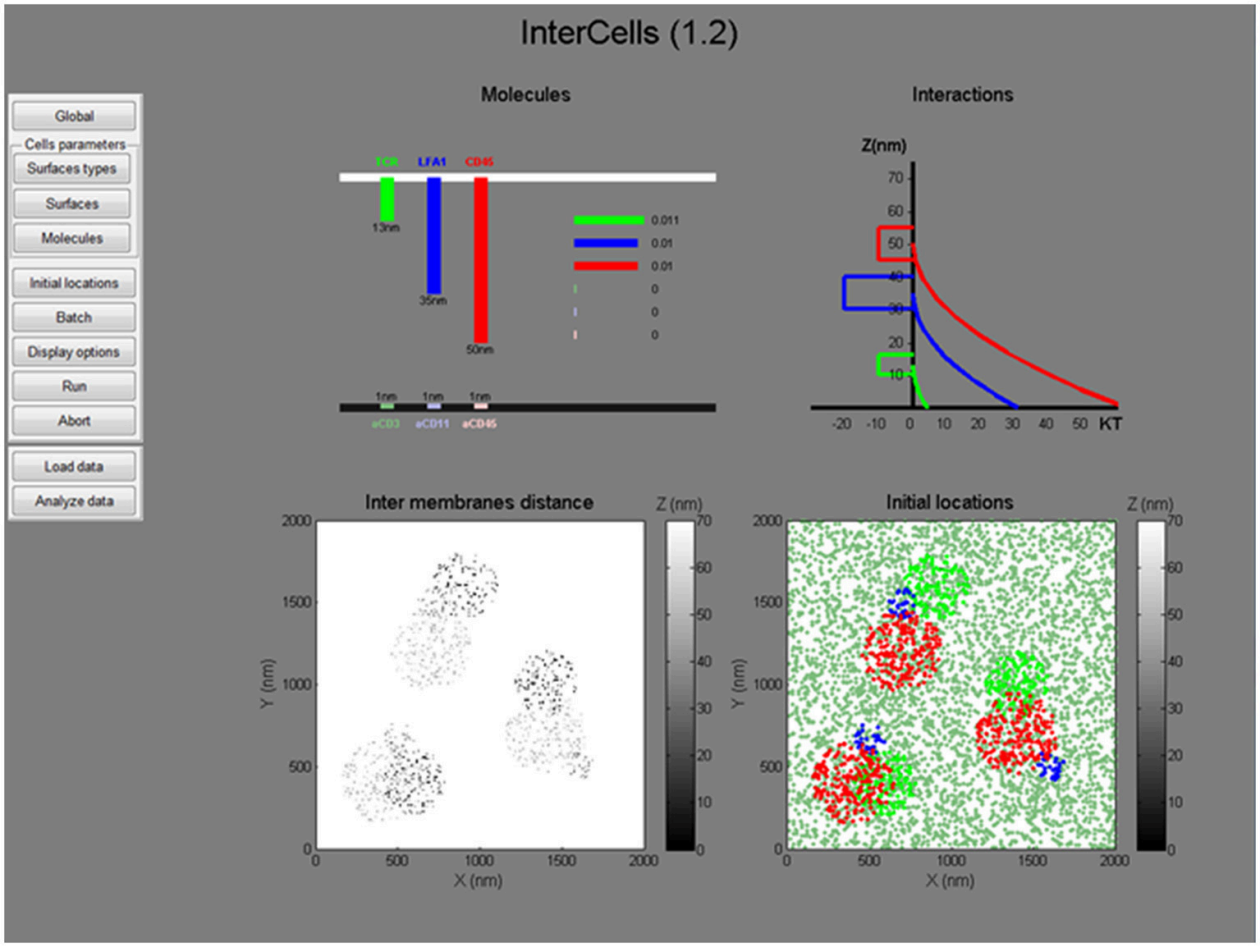
The graphical user interface. A snapshot of the graphical user interface (GUI) of the simulation. The GUI includes dynamic menus for setting all simulation parameters and a live interface for presenting the simulated parameters (top) and initial conditions (bottom). All menus, parameters and operation instructions are provided in the User's Manual in the Supplemental Information.

For testing the robustness and dynamics of the patterns that we have detected, we turned to the modeling and simulation of the cell interfaces. The simulation is described in details in the Materials and Methods and in the User's Guide (provided in the Supplemental Information). Briefly, the simulation employs physical modeling of the PM of the interacting cells and of the molecular interactions (Figure 3, and below). The simulation structure is described in Figure 4, the simulation process is described in Figure 5 and its GUI is shown in Figure 6.

An important feature of our agent-based Monte-Carlo simulation, is its ability to integrate experimental measurements at the single molecule level (as in Figure 2). Such data can be integrated as initial conditions for the simulation, or as dynamic physical constraints [as previously demonstrated (18)]. Here, we demonstrate the setting of initial conditions by cropped data from the footprints of cells, imaged by SMLM (Figure 2). Specifically, coordinates were taken for TCRζ molecules (in green). To complete the initial conditions, the initial coordinates of CD11 and CD45 molecules were manually determined via the available tools of the simulation in the GUI.

In our simulation, the plasma membrane of the interacting cells are modeled as grids where molecules, modeled as agents, diffuse and interact within and across the grids (Figures 3A,B). The simulation included a model that captured the energetic of the PMs of interacting T cell and APC (26) (Figure 3C). Specifically, the simulation balanced forces due to attractive and repulsive interactions. Specific attraction occurred between the TCR and αCD3 and self-clustering of CD11 and TCR molecules. Non-specific attraction affected the molecules at the T cell PM by the PLL. Repulsive interactions occurred between the molecule (and esp. for the bulky glycoprotein) and the coverslip (Figure 3C). The PM underwent thermal fluctuations during the simulation. The positions of the molecules were updated in each step of the simulation. The simulations included 10,000 steps of 400 × 400 pixels of 10 nm each and took ~5 min (~100 s in cell time) each, using a PC (i7 quad processor). Simulated parameters are detailed for the interacting molecules (Table S1).

**Figure 7:**
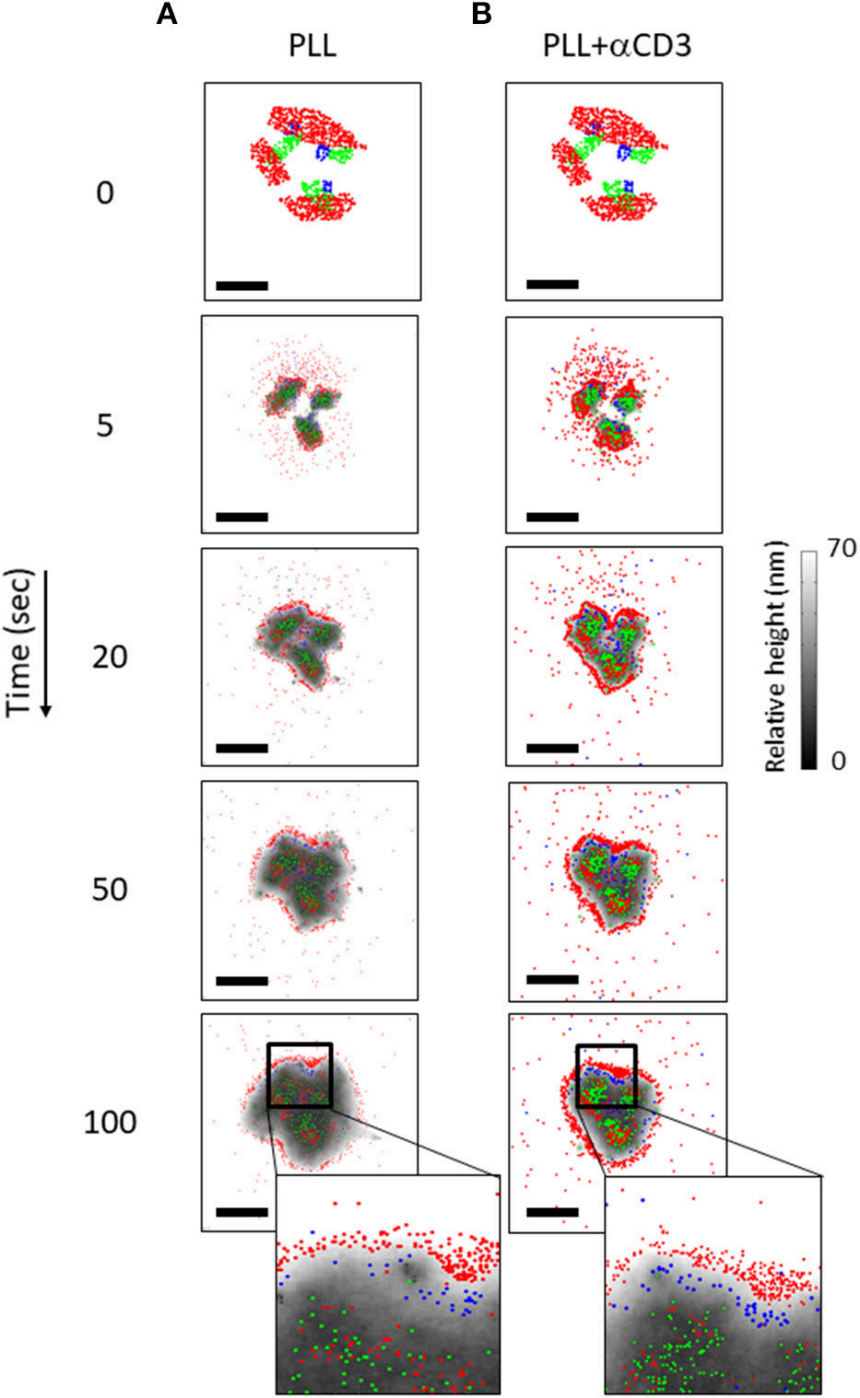
The dynamics of molecular patterning in simulations. The dynamic evolution of molecular patterning of TCRs (green), CD11 (blue) and CD45 (red) at the cell interface. Simulated results are shown as a function of time for individual cells on either a PLL–coated coverslip **(A)** or on an αCD3-coated coverslip **(B)** along time. The simulations start from user-defined initial conditions (top raw) that are set for the simulated molecules. Membrane height is marked by variable gray levels. Bars−1 μm.

The simulation resulted in a redistributed pattern of molecules that was embedded within the interface, and evolved over time (Figures 7A,B, Movies M1, M2). Strikingly, the simulations could recreate realistic patterning of CD45 molecules around the evolving TCR clusters. CD11 molecules were distributed in between TCR clusters and CD45. These results correlated well with experimentally imaged positions of these molecules on either PLL- or αCD3-coated coverslips (compare left and right columns in Figures 7A,B with Figures 2A,B).

### The effect of simulation parameters on the molecular patterning

Multiple parameters could affect the resultant molecular patterns that we observed. Such parameters include the initial conditions of molecular placement (e.g., in Figure 7 at *t* = 0); the density of the molecules; their diffusion coefficient and their interaction potential. Thus, we repeated the simulations shown in Figure 7, yet with modifying one of the described parameters in each simulation. Recent publications showed that TCRs are clustered in microvilli (27) that form early contacts (18, 28). Hence, we started with changing the initial placement of CD11 and CD45 molecules in relation to TCR clusters. The cells were attached to a coverslip coated with PLL and αCD3, for engaging the TCRs. Figure S1 shows the results for applying the initial conditions as in Figure 7B (Figures S1A–C), a diffused pattern of CD45 (Figures S1D–F) or a diffused pattern of both CD45 and CD11 (Figures S1G–I). The variability in initial conditions was applied to simulations that either included molecular self-clustering of TCR and CD11 (Figures S1B,E,H) or did not include such self-clustering (Figures S1C,E,I). Strikingly, the molecular patterning under all conditions showed the mutual patterning of TCRs, CD11 and CD45, as in Figure 7B an in our experiments (Figure 2B). As expected, TCR and CD11 were more diffused within these mutual patterns when the simulations did not include self-clustering of these molecules (Figures S1C,E,I). Our results indicate the robustness of the mutual patterning of TCR, CD11 and CD45 to variations in initial molecular placements and to their self-clustering.

We next conducted a sensitivity analysis of molecular patterning to variations in the density of CD45 (Figure S2A), the diffusion coefficient of the molecules (namely, TCR, CD11 and CD45; Figure S2B), and the interaction potential of CD45 (Figure S2C). Parameter values were taken as half, equal or twice the values that were chosen in the simulation shown in Figure S1B. The mutual patterning of TCR, CD11 and CD45 was robust to most of the conditions. Still, the following differences can be observed for the different conditions. For instance, the CD45 outer ring became relatively thicker with the increase of CD45 concentration (Figure S2A). The mutual shape of the TCR, CD11 and CD45 became more diffused and occupied a bigger area with the increase in the diffusion coefficients of the molecules (Figure S2B). Last, we observed a less diffused pattern of CD45 when its spring constant became stronger (Figure S2C).

### Modeling and simulation of molecular patterning at the T cell-APC IS

**Figure 8:**
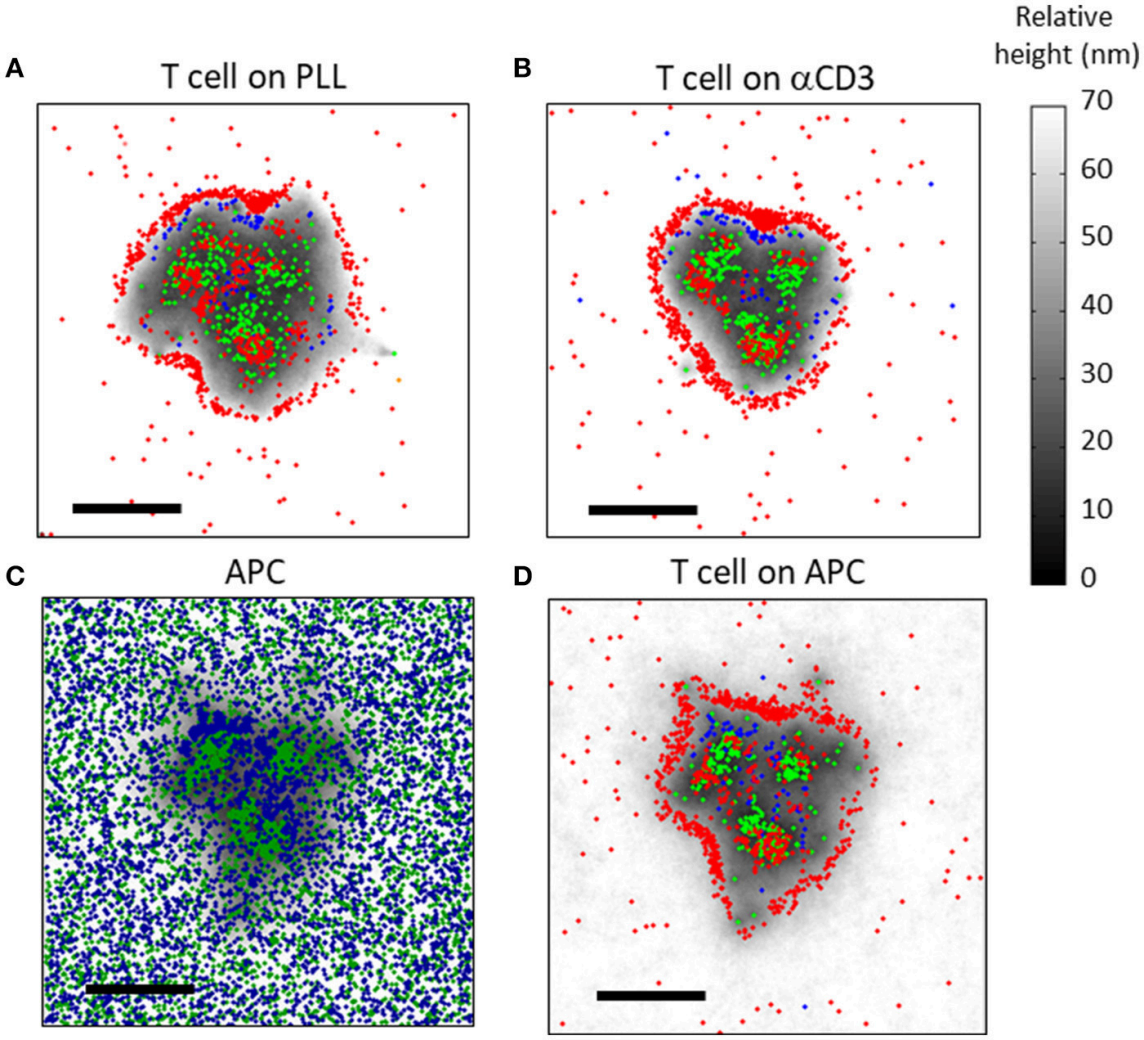
Simulation results of molecular patterning at the immune synapse **(A,B)** The molecular patterning of TCRs (green), CD11 (blue) and CD45 (red) at the cell interface, on either **(A)** a PLL-coated or **(B)** an αCD3-coated coverslip. **(C,D)** The molecular patterning at the T cell-APC immune synapse. **(C)** ICAM (blue) and pMHC (green) molecules are shown at the PM of the APC. **(D)** TCRs (green), CD11 (blue) and CD45 (red) are shown at the PM of the T cell. Membrane height in panels **(C,D)** is marked by variable gray levels. Bars−1 μm.

Next, we studied the effects of the patterning in simulated physiological interface between T cells and APCs. Nanoscale imaging of T cells and APCs is technically complicated, yet is readily accessible to our modeling and simulation. Here, we included mobile ligands at the PM of the APC, namely pMHC and ICAM. These molecules exerted specific attraction forces on the TCR and CD11 molecules (respectively) as they diffused at the PM of the opposing T cell. The PM of the APC was given similar physical properties to the PM of the T cell (as detailed in Table S2). The molecular positions at the T cell PM were set manually as initial conditions, and were kept identical for the simulations on APCs (Figures 8C,D) and on coverslips coated with either PLL (Figure 8A) or αCD3 (Figure 8B). In this way, results could be directly compared across different interfaces.

Our simulations showed that the mutual patterning of CD11, TCRs and CD45 occurred not only on coverslips, but also at the PM of T cells conjugated to APCs (Figures 8C,D). Corresponding patterning of pMHC and of ICAM molecules appeared at the PM of the APCs (Figure 8C). Interestingly, CD11 was less self-clustered in such interfaces in comparison to the cell interface with αCD3-coated coverslips (Figure 8B).

Under physiological conditions, APCs typically carry only a small fraction of cognate peptides. Thus, we repeated our simulations for interface of T cells with APCs, while considering only 1% of cognate peptides (Figures S3A,B). As expected, the interface was not as tight as for the previous simulation (compare height levels with Figures 8C,D). Importantly, the molecular patterning of TCR, CD11 and CD45 seemed more diffused and their segregation was less pronounced.

Another important physiological condition is the translocation of TCR molecules toward the center of the IS 21 (29, 30). To simulate this process, we created an interactive tool within the software. Using this tool, we set a target coordinate for TCRs translocation at the center of the interface and set a constant velocity of 19 nm/s (29) toward the center to all TCRs. Along with translocation, we assumed TCR diffusion but no self-clustering, in order not to hinder its mobility further. Expectedly, the TCRs concentrated at the center of the IS, while a relatively pronounced and well-segregated CD45 ring formed at the periphery (Figures S3C,D). As before, CD11 molecules localized between the segregated TCRs and CD45 molecular patterns.

**Figure 9:**
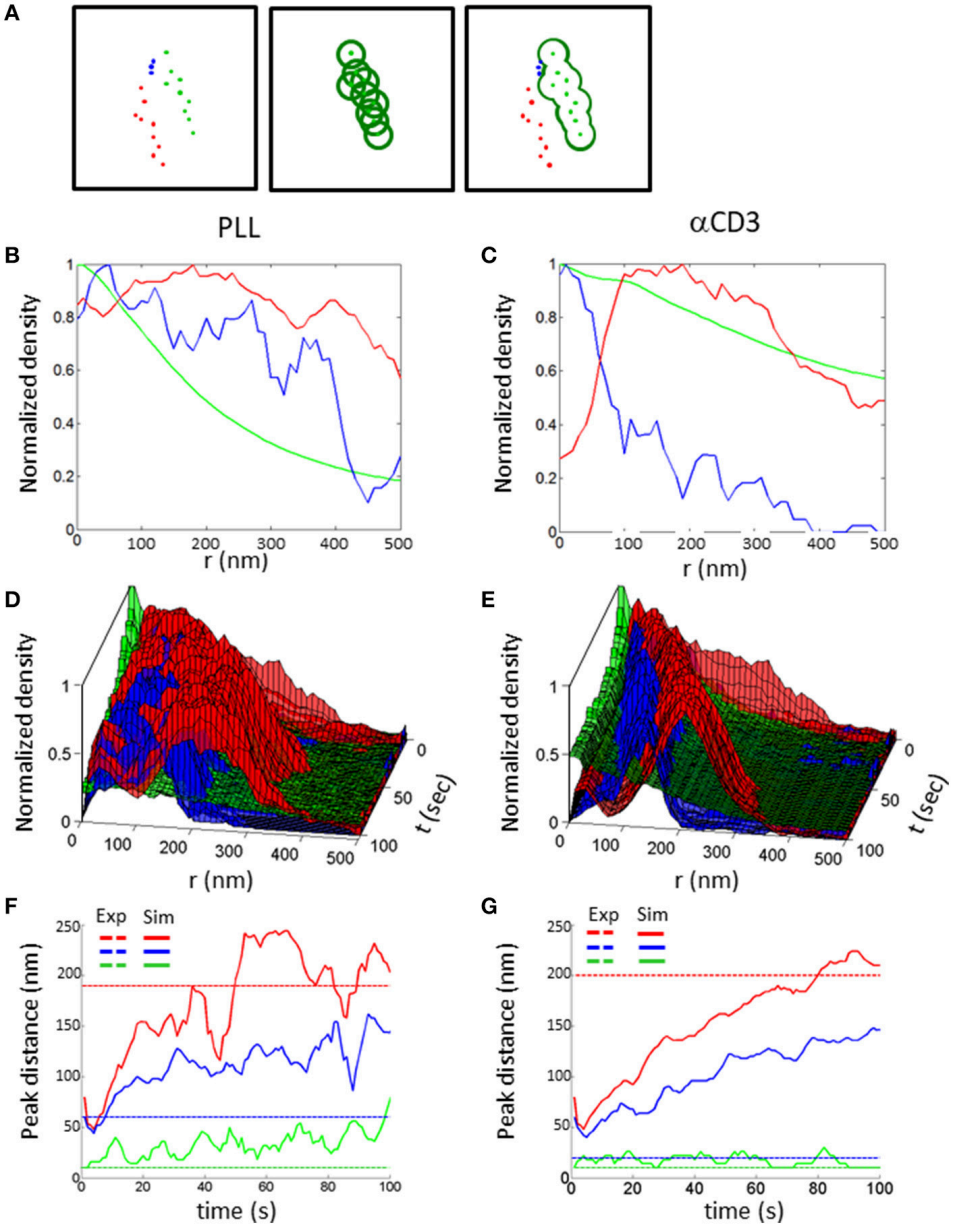
Topology analysis of molecular patterning in experiments and simulations. **(A)** Topology analysis of molecular densities of different species (red and blue dots) relative to a cluster of reference (green dots). The cluster perimeter is defined by consecutive dilations. **(B,C)** Results of the analysis of molecular patterning in experimental results on either **(B)** a PLL–coated coverslip or **(C)** on an αCD3-coated coverslip. **(D,E)** Results of the analysis of molecular patterning in simulated results, on either **(D)** a PLL–coated coverslip or **(D)** on an αCD3-coated coverslip. **(F,G)** The shift in the peak of the molecular distributions of the topology analyses, on either **(F)** a PLL–coated coverslip or **(G)** on an αCD3-coated coverslip. Experimental results are shown as dashed colored lines while simulated results are shown as continuous colored lines.

To quantitatively assess the mutual patterns of TCR, CD11 and CD45, we introduced a topological analysis (see details in the Materials and Methods and in Figure 9A). This analysis related the density of CD11 and CD45 molecules to individual TCR clusters. The density of the molecules as a function of the distance from TCR clusters is shown in Figures 9B–E. The results of the topological analysis of our experimental results (in Figure 2) clearly show the hierarchical ordering of TCR clusters at the center, surrounded consecutively by CD11 and CD45 molecules (Figures 8B,C). Moreover, the evolution of this pattern could now be captured using our simulated results on either PLL- or αCD3-coated coverslips (Figures 9D,E). As expected, the self-clustering of TCRs (the peak height of the green manifold) was higher, and more persistent for αCD3-coated coverslips relative to PLL-coated coverslips. Strikingly, the mutual patterning of TCR, CD11 and CD45 occurred within a few 10 s of seconds from the start of the simulations. The mutual patterning of CD11 and CD45 from TCR can be further compared between the experimental data and the simulated results (Figures 9F,G). Our simulations captured the shift in the peak of the molecular distributions of CD45 relative to the TCRs on both PLL (Figure 9F, red lines), and on αCD3 coated coverslips (Figure 9G, red lines). The separation of CD11 was captured more accurately on PLL-coated coverslips than on aCD3-coated coverslips (Figures 9F,G, blue lines). Thus, our simulations now set the stage for seeking parameters that would minimize the differences between the density distributions of the molecules under study (18).

## Discussion

In this work, we introduce “InterCells”—a generic agent-based Monte-Carlo simulation of intercellular interfaces in molecular detail. Our study focused on dynamic molecular patterning at the early IS, as an important example of a dynamic intercellular interface. The study combined three-color SMLM imaging of fixed CD4^+^ T cells on functionally coated coverslips, as well as modeling and simulations of the IS of such CD4^+^ T cells with APCs. Our imaging and simulation showed an intricate patterning of TCRs, glycoproteins (e.g., CD45) and integrins (e.g., CD11) at the PM of the interacting T cells. In the detected patterns, clusters of CD11 localized in between segregated clusters of TCR and of CD45. Such patterning has been instructed by diffraction limited microscopy (12), recent detection of segregation of TCRs and CD45 molecules in early contacts (18), and by preliminary simulations. To our knowledge, such mutual patterns have not been observed at the nanoscale before and thus, have not been related to the macroscopic “bull's eye” patterns detected at the IS (10, 21).

To test the robustness of the detected pattern, we simulated a range of relevant interfaces, including coverslips with different coatings, different sets of initial conditions, and ISs with APCs. Varying such conditions in the simulation can be readily achieved through the design and the GUI of the simulation. Notably, our agent based simulation allows for the seamless integration of experimental data at the single molecule level, as captured by PALM and dSTORM. We have previously demonstrated the use of SMLM data as constrains for setting hybrid simulations (18). The results of such simulations can be directly compared to experimental results (18). The robustness of the molecular segregation between TCR, CD11 and CD45 clusters, which persisted in all simulations, indicates that it is driven by mechanical forces acting between molecules and the opposing surfaces of the IS. Notably, our simulation did not include translocation of molecules, such as TCRs or integrins, across the IS (31). Such translocation plays a role in the spatial sorting of newly appearing clusters at the cell periphery in the mature IS, while our simulation and imaging focused on relatively less mature ISs.

Multiple simulation tools have been developed to study molecular interactions in the cell, such as signaling pathways and enzymatic reactions. Such modeling often assumes complete molecular mixing via ordinary differential equations (ODEs) (32), or the use of cell automata with cell compartmentation that could average out critical spatial variations in local concentration of signaling proteins. The virtual cell [Vcell; (33)] allows for solving partial differential equations (PDEs) and ODEs, and the integration of spatial constraints from 2D and 3D optical microscopy. Still, such simulations cannot account for molecular heterogeneities and non-synchrony that are inherent to stochastic processes of molecular diffusion and interaction within cells. Such heterogeneities can be captured by Monte Carlo simulations of finite numbers of interacting molecules that are embedded in realistic models of cellular compartments [e.g., Smoldyn (34) and MCell (35)]. Specifically, MCell contains extensive simulation tools, including the generation of arbitrary meshes through integration with a powerful graphical package (Blender), the simulation of cytosolic proteins, allowing stochastic state transitions of molecules, various mobility states including diffusion and drift and running batches for scanning parameters. Notably, MCell is not designed to account for dynamically changing meshes. In contrast to MCell, InterCells is currently more modest in its flexibility and in its integration of advanced features and tools. For instance, multiple dynamic processes, such as molecular endocytosis and recycling are currently lacking and will become available in an upcoming update of the simulation. Also, it is currently limited to simulating membrane proteins, while cytosolic proteins will be integrated, but will not be explicitly simulated as diffusing agents in the 3D environment of the cytosol. Still, our simulation specializes in capturing complex and dynamic interactions and pattern formation in intercellular interfaces. It focuses on surface molecules interacting in a dynamic, fluctuating surfaces. To our knowledge, the integration of SMLM data into cell simulations and the effects of embedded molecules on the cells' surface are important features that have not been attempted in current simulations.

Our simulation has been designed as an accessible tool to non-experts. It operates on a PC with a standard (i7 quad) processor. It is coded in Matlab, in a modular structure that can be easily expanded to include additional membrane and cortical structures, such as cortical cytoskeleton, membrane bound proteins, channels, etc. Still, expansion of the simulation to whole cells will require much stronger computational power than is currently employed. It integrates a wide range of physical parameters of simulated entities (membranes, molecules) that are accessible via an intuitive GUI. We provide multiple analysis tools, including univariate and bivariate PCFs (36), clustering algorithms and the topological analysis demonstrated here (Figure 9). Additional analyses of relevance may include Minkowski functionals (37), conditional second order PCFs (13), and more. Our simulation enable batch runs for scanning systematically values of parameters of choice. The results of such batch simulations can be presented graphically using our statistical analyses tools. The user can then quantitatively compare the results of such statistics to experimental results, for further refinement of the simulation. We have recently demonstrated this approach to study inaccessible properties of the PM (e.g., its rigidity and its ligand density) (18). The further integration of iterative simulations with sensitivity analyses such as the Sobol method could enable more systematic evaluation of the wide parameter space of our agent-based simulation.

We believe that InterCells, esp. with its upcoming tools, will allow the study of molecular patterning at cell surfaces and interfaces in a wide range of cases. We provided in the User's Manual a second example, demonstrating how InterCells can be employed to quantify the effects of molecular trapping and self-clustering on molecular organization at the PM. Additional cell interfaces that can be studied using InterCells may include cell junctions between cells in a tissue, the evolution of interfaces in development, neuronal synapses, immune synapses of multiple types, and under various experimental conditions, and more.

To conclude, we provide here a generic simulation of intercellular interfaces. The simulation was applied to nano-scale pattern formation at the IS, which was resolved by three-color SMLM. The detailed simulations combined data from SMLM imaging, coarse-grained physical model PM of the interacting cells, and simulative data from multiple Monte-Carlo simulations. During this process, the simulation has proved to be an invaluable predictive and hypothesis generating tool. It further provided an elaborate test of our physical understanding of molecular patterning at the IS and of the forces behind it. The iterative application of novel experimental tools and modeling could provide critical feedback to future experiments and the adaptation of working models; thus, in this case, enhancing our mechanistic understanding of early T cell activation. Our simulations are modular, flexible and accessible, such that they can be employed for studying a wide range of intercellular interfaces and molecular interactions within.

## Methods

### Sample preparation

Jurkat E6.1 cells and such cells stably expressing TCRζ-Dronpa were available for this study from a previously published work (16). Positive expression was routinely monitored using fluorescence microscopy. For three-color, SMLM TCRζ-Dronpa were immunostained with antibodies: 1. αCD45-Alexa647 conjugated (BioLegend, 304056); 2. αCD11 (LFA1) primary (BD Pharmingen, 555378) and αMouse secondary antibody labbled with Alexa568.

Cells were dropped onto glass coverslips coated with 0.01% poly-L-lysine (Sigma) with or without following coating αCD3 (UCHT1, eBioscience 16-0038-85). The cells were incubated at 37°C for a specific spreading time on the coverslips of 1.5 min. After this time the cells were fixed with 2.4% Paraformaldehyde for 30 min at 37°C. Combined SMLM (PALM-dSTORM) imaging was performed in a dSTORM buffer (50 mM TRIS pH = 8, 10 mM NaCl, 0.5 mg/ml glucose oxidase, 40 μg/ml catalase, 10% glucose, 10 mM MEA).

### PALM and STORM microscopy

Three-color SMLM (combined PALM/dSTORM) imaging was performed using a total internal reflection (TIRF) microscope (TI-E, Nikon). Imaging in TIRF mode served to visualize molecules at the PM of spreading cells in close proximity to the coverslip (up to ~100–200 nm). PALM images were analyzed using the N-STORM module in NIS-Elements (Nikon) or a previously described algorithm (ThunderSTORM) (38) to identify peaks and group them into functions that reflect the positions of single molecules (14). PALM acquisition sequence typically took ~5 min for three channel imaging at 50–100 frame/s. Custom algorithms were then applied for statistical characterization of the SMLM images of the detected molecules (see Supplementary Information for further details). The fluorescent proteins were imaged sequentially in the different channels using dedicated emission filters that minimized cross talk between the channels. Photoactivation illumination at 405 nm was changed over the imaging sequence of fixed cells. Drift compensation and channel registration were performed using dedicated algorithms in ThunderSTORM.

## Detailed molecular simulation

### Modeling approach and structure of the simulation

Here, we take a reductionist approach for modeling, aiming to explain complex spatio-temporal patterns of molecular organization at intercellular interfaces. All simulation files are available online on Github (https://github.com/ShermanLab/InterCells). These files should be downloaded to the User's computer under a directory that can be accessed by Matlab.

#### Requirements

For ease of use, the simulation Basic computational power, employing a standard PC (with an i7 processor). It is coded in Matlab (MathWorks). The structure of the simulation is depicted in Figures 4,5 and is explained in detail in the User's Manual (provided in the Supplemental Information).

#### Input

Input parameters include parameters that describe the physical properties of the interacting surfaces and of the molecules that interact within and across the interfaces. The parameters are typically extracted from experimental measurements, on molecular interactions that govern the signaling cascade (39, 40). In the case of hybrid simulations, initial conditions are set by single molecule data on molecular positions and their state from SMLM imaging (see User's Manual). Benchmark runs for testing a range of predetermined parameters. Such benchmark runs have previously served for critical evaluation of mechanistic models of T cell activation (24) and for studying the effects of variations in critical physical parameters on molecular patterning at the IS (18).

#### Simulation core

The simulation includes detailed models of relevant stochastic processes, including reaction-diffusion processes and relevant force fields. The details of the simulation algorithm are provided in separate sections below. Briefly, in our simulation we assume specific Hamiltonians of a quasi-equilibrium system and with mean-field approximations. The simulation relies on hierarchical levels of simplification. Continuous entities that are not the focus of the simulations are “coarse-grained.” Such entities include lipids in the PM and water molecules, and are not specifically described in the simulation. In contrast, protein molecules of interest are described individually. The simulation algorithms is realized using “importance sampling” Monte-Carlo simulations (41). Molecular identities are maintained for the reactant molecules of interest. Metropolis criterion is applied to determine the transition probability between consecutive configurations.

#### Outputs

Quantifiable readouts of the numeric simulations include the position and state of individual proteins, the morphology of the PM and their energetics. Visualization tools are provided for showing the simulation results. For instance, live evolution of molecular patterning is provided during the simulation run. The patterns can then be shown for each step individually, or as a movie.

#### Analyses

Here we integrated multiple statistical tools for quantitative analyses and interpretation of the results. Our tools include clustering algorithms and second-order statistics (16, 36), and the topology analysis (Figure 3). These tools are important for the quantitative comparison between results from experiments and from simulations. Moreover, the analyses provide a critical feedback for generating experimentally testable hypotheses and the adaptation of working models in an iterative way. In fact, our imaging in this study was instructed by early simulative results that indicated the mutual patterning of TCR, CD11 and CD45.

#### Simulation setup

The simulations are based on a rectangular grid, of a size of few microns. The array is made of square 10 nm pixels. We used periodic boundary conditions (molecules that exit on one side appear on the opposite side). The initial height (*z*) of the membrane is set to 70 nm. The PM height in pixels that accommodate either TCR or CD11 molecules are set to the molecular height. The *z*-value of each pixel changes randomly at every iteration by Δ*z* that has a normal distribution with σ = 1 nm, according to the Metropolis criterion.

A specific limitation of our simulation to the number of simulated molecules originates from the occupancy of only one molecule (regardless of its species) in a single pixel. Thus, considering a pixel size of 10 nm and a rectangular grid of 1 × 1 μm^2^, a limit of 10 K molecules can be simulated. Larger grids are often needed to show complex molecular patterns within a cell footprint. Thus, we often simulated tens of thousands of molecules within grids of 400 × 400 pixels. Such grids were chosen to include a region of interest of a cell footprint with an area of 4 × 4 μm^2^ (i.e., each pixel representing an area of 10 × 10 nm). Such a size should leave a wide enough margin (e.g., ~50–100 pixels), such that boundary effects are minimized. Such simulations took ~15 min using a PC with a standard (i7 quad) processor. Acceleration of the simulation can be improved via operating parallel computing, computation via GPUs and more. A bigger grid size minimizes the effect of the boundary, yet requires longer (actual) simulation time, computational power and memory. Similar consideration may restrict the iteration time, overall simulation time, the save rate and the number of runs (Table S3). While other simulations, such as MCell, can accommodate millions of molecules and states, they require compartmentation of the simulated space for efficiently running.

We simulated multiple different types of proteins, as follows. TCRs behave as binding proteins to immobile ligands (αCD3) on a coverslip or to mobile pMHC molecules at the PM of APCs. CD11 may bind ICAM at the PM of APCs. The *z* coordinates of the TCRs and CD11 are kept at 13 nm and at 35 nm, respectively, throughout the simulation runtime. The molecules, and esp. bulky CD45 molecules, act as repulsive springs. Non-specific binding occurs between all molecules and the PLL coating of coverslips. The numbers of simulated molecules remains constant throughout the simulation. All simulated parameters are detailed in Tables S1–S3.

### Monte Carlo simulations

#### Simulation energetics

In the simulations we used the Hamiltonian *H* = *H*_*int*_ + *H*_*el*_, to calculate the energetics of the overall interactions between the T cell membrane and the coverslip (represented by the term *H*_*int*_) and the elasticity of the T cell membrane (represented by the term *H*_*el*_). The interaction part, *H*_*int*_, is defined as:

(1) $$\begin{array}{l} H_{int}=\sum_{i}\left(\delta_{1,mol_{i}}\delta_{1,lig_{i}}\right)V_{mol-lig}\left(z_{i}\right)+\delta_{1,mol_{i}}V_{mol}\left(z_{i}\right) \end{array}$$

where,

(2) $$\begin{array}{l} \delta_{1,X_{i}}=\{\begin{array}{c} 1,\;if\;a\;molecule\;of\;type\;X\;exists\;in\;pixel\;i\\ \;0,\;otherwise\; \end{array} \end{array}$$

Single pixels from any surface (i.e., either a PM or a coverslip) can accommodate only one molecule at a time. The interaction potential of the molecule with its ligand, *V*_*mol*−*lig*_, is defined as:

(3) $$\begin{array}{l} V_{mol-lig}\left(z_{i}\right)=\{\begin{array}{c} U_{mol-lig},\;|z_{i}-l_{mol-lig}|\;<Interaction\;range\\ \;0,\;elsewhere\; \end{array} \end{array}$$

where *U*_*mol*−*lig*_ is the interaction strength of a molecule and its ligand, *l*_*mol*−*lig*_ is the length of an engaged molecule-ligand conplex. *z*_*i*_ is the inter surface distance at pixel *i*. The width of the molecule-ligand potential is set and its depth are set according to published results (see Table S3). The repulsion potential of the molecule is defined as:

(4) $$\begin{array}{l} V_{mol}(z_{i})=\{\begin{array}{c} k_{mol}{\left(z_{i}-l_{mol}\right)}^{2},\;z_{i}<\;l_{mol}\\ \;0,\;z_{i}>\;l_{mol}\; \end{array} \end{array}$$

The physical parameters of k_mol_, the compressional stiffness of the molecule and *l*_*mol*_, the length of the uncompressed molecules, are detailed in Table S1.

The elastic part of the Hamiltonian, *Hel*, is defined as:

(5) $$\begin{array}{l} H_{el}=\sum_{i}\frac{\kappa }{2a^{2}}{\left(\Delta_{d}z_{i}\right)}^{2} \end{array}$$

where κ = κ_1_·κ_2_/(κ_1_ + κ_2_), is the general effective bending rigidity of two membranes. In this case, the bending rigidity is effectively κ ≈ κ_1_, since κ_2_ >> κ_1_ and is simulated at different values. The lattice constant, a, is 10 nm and _*d*_*z*_*i*_ = *z*_*i*1_+*z*_*i*2_+*z*_*i*3_+*z*_*i*4_−4*z*_*i*_, (where *i*1, *i*2, *i*3, *i*4 are the indices of the four nearest neighbors of pixel *i*).

#### Simulations dynamics

The simulation propagates in time by iterations of 0.01 s. In every iteration all molecules attempt to hop to one of the neighboring pixels according to their diffusion coefficient. The hopping attempts of the molecules are accepted or rejected according to the following rules:

1. The target pixel is not occupied.
2. The probability of acceptance is according to Metropolis criterion is:

at an old pixel:

(6) $$\begin{array}{l} P(old\;state\to free)=\{\begin{array}{c} 1\Delta E<0\\ \exp\left(-\Delta E\right)\Delta E>0 \end{array} \end{array}$$

and at a new pixel:

(7) $$\begin{array}{l} P(free\to new\;state)=\{\begin{array}{c} 1\Delta E<0\\ \exp\left(-\Delta E\right)\Delta E>0 \end{array} \end{array}$$

While

(8) $$\begin{array}{l} P\left(attempt\;accepted\right)=P\left(old\;state\to free\right)\times P\left(free\to new\;state\right) \end{array}$$

3. If more than one molecule attempted to hop to the same pixel, the molecule with the highest energy gain will hop.
4. The height, *z*, of each pixel of the surface is changed randomly by Δ*z*, that has a normal distribution with σ = 1 nm and according to Metropolis criterion. The value of σ is set by receiving 40–50% of acceptance of the membrane attempts 27.

### Topology analyses

The topology analysis measures the conditional density of molecules from a spatial reference set by clusters of a chosen molecular type. In the example presented in Figure 9A, the cluster of reference is set by green molecules. Next, circles are placed around each green molecule (middle panel), and we consider the perimeter of their unified area. The densities of the other molecules (namely, red or blue points in our example) can now be calculated on this perimeter. The consecutive operation of these steps with growing radii from the molecules yields the Minkowski perimeter functional (37). The conditional densities of the molecules are then calculated for the growing perimeters, as shown in Figures 9B–E. Last, the density of the molecules of reference (here, green dots) is determined and presented by its univariate PCF as a function of the perimeter radius.

## Author contributions

ES supervised research; ES, YN-O designed research; YN-O developed and performed simulations; YR and JS developed reagents and performed imaging experiments research; ES, YN-O wrote the paper.

### Conflict of interest statement

The authors declare that the research was conducted in the absence of any commercial or financial relationships that could be construed as a potential conflict of interest.
