# Supplementary material for: InterCells: A Generic Monte-Carlo Simulation of Intercellular Interfaces Captures Nanoscale Patterning at the Immune Synapse
Source: Front Immunol. 2018 Sep 11;9:2051. doi: 10.3389/fimmu.2018.02051 (PMC6141710; doi:10.3389/fimmu.2018.02051)
Supplement: Supplementary file 1 [file Table_1.pdf]

Table S1. Default molecules' parameters												
	**T-cell**			**Coverslip**			**APC**		**Lipid bilayer**		**Units**	**Refs**
Name/Colour	TCR	LFA-1	CD45	aCD3	aCD11	aCD45	pMHC	ICAM	pMHC	ICAM		
Type number	1	2	3	1	2	3	1	2	1	2		
sizes												
Vertical size	13	35	50	0	0	0	0	0	0	0	nm	[26]
Lateral size	10	10	10	10	10	10	10	10	10	10	nm	[26]
Area	1	1	1	1	1	1	1	1	1	1	pixels	[26]
potentials												
Potential width	6	10	10	0	0	0	0	0	0	0	nm	[26]
Binding bottom	10	30	45	0	0	0	0	0	0	0	nm	[26]
Binding top	16	40	55	0	0	0	0	0	0	0	nm	[26]
Binding strength***	-10	-20	-10	-10	-10	-10	-10	-20	-10	-10	KT	[26]
k spring**	0.1	0.1	0.1	0	0	0	0	0	0	0	KT/nm^2	[26]
Force membrane to molecule height	Yes	Yes	No	No	No	No	No	No	No	No	Yes/No	
Diffusion and distributions												
Diffusion constant**	0.01	0.01	0.01	0	0	0	0.01	0.01	0.01	0.01	$\mu m^2/sec$	[26]
Global density**	300	300	300	300	300	300	300	300	300	300	$\#/\mu m^2$	[18]
Cluster density*	1000	1000	1000	1000	1000	1000	1000	1000	1000	1000	$\#/\mu m^2$	[19]
Density of clusters*	0.8	0.8	0.8	0.8	0.8	0.8	0.8	0.8	0.8	0.8	$\#/\mu m^2$	
Dynamics												
Self-clustering	Yes	Yes	No	No	No	No	No	No	No	No	Yes/No	
Self-clustering binding range	10	10	10	0	0	0	10	10	10	10	nm	
Self-clustering P_{on}	0.995	0.995	0	0	0	0	0	0	0	0	[0 1]	
Self-clustering P_{off}	0.005	0.005	1	1	1	1	1	1	1	1	[0 1]	
Transport												
Use transport	Yes	No	No	No	No	No	No	No	No	No	Yes/No	
Transport speed	19	0	0	0	0	0	0	0	0	0	nm/sec	[29]

Comments:
* - sensitivity analysis is provided in Fig. S1
**- sensitivity analysis is provided in Fig. S2
*** - sensitivity analysis is provided in Fig. S3

Table S2. Default membranes' parameters						
	T-cell	APC	Coverslip	Lipid bilayer	Units	Refs
Rigidity						
Rigidity	25	25	10^6	10^6	KT	[26]
Minimum rigidity	25	25	10^6	10^6	KT	[26]
Maximum rigidity	100	25	10^6	10^6	KT	[26]
Local rigidity	No	No	10^6	10^6	Yes / No	
Diffusivity						
Diffusivity	1	1	0	1	[0 1]	
Minimum diffusivity	1	1	0	1	0	
Maximum diffusivity	1	1	0	1	1	
Local diffusivity	No	No	No	No	Yes / No	
Height						
Z_0	70	0	0	0	nm	
Minimum height	10	0	0	0	nm	
Maximum height	100	0	0		nm	
Sigma dz	1	1	0	0	nm	[26]

Table S3. Default global parameters		
Array		Units
size x	400	pixels
size y	400	pixels
Pixel size	10	nm
Times		
Iteration time	0.01	sec
Simulation time	100	sec
Experiment frame rate	2.5	sec
Save rate	100	iterations
Dynamics		
Metropolis steps	2	1,2
Stick time	100	sec
Poly-L-lysine		
Binding strength	-4	KT
Use poly-L-lysine	Yes	Yes / No
Number of runs		
Number of runs	1	
Membrane domains		
Use adhesive domains	Yes	Yes/No
Adhesion strength	-4	KT
Circular domain radius	20	nm
Actin		
Use actin	No	Yes/No
Actin rigidity	100	KT

Figure S1

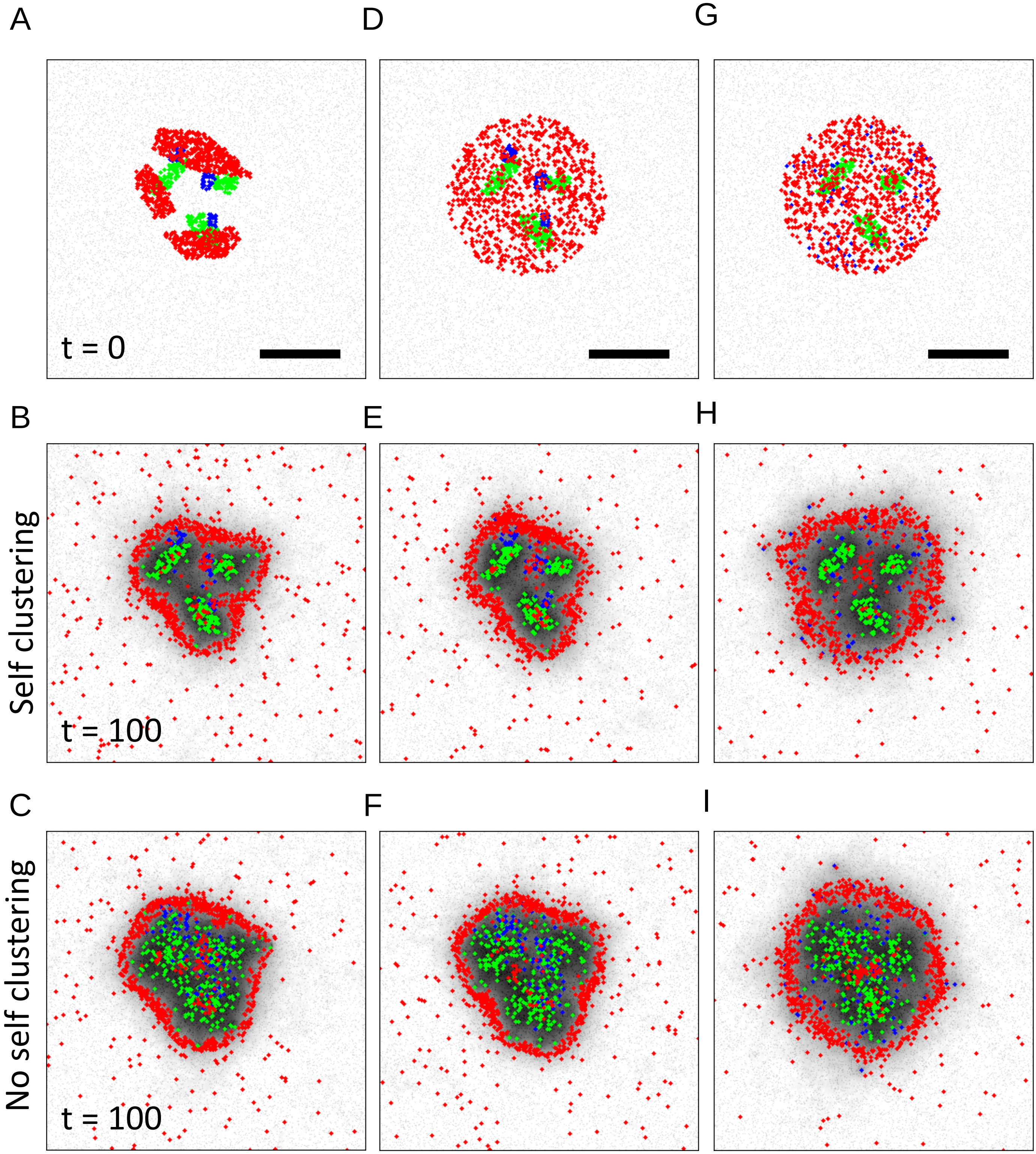

Figure S2

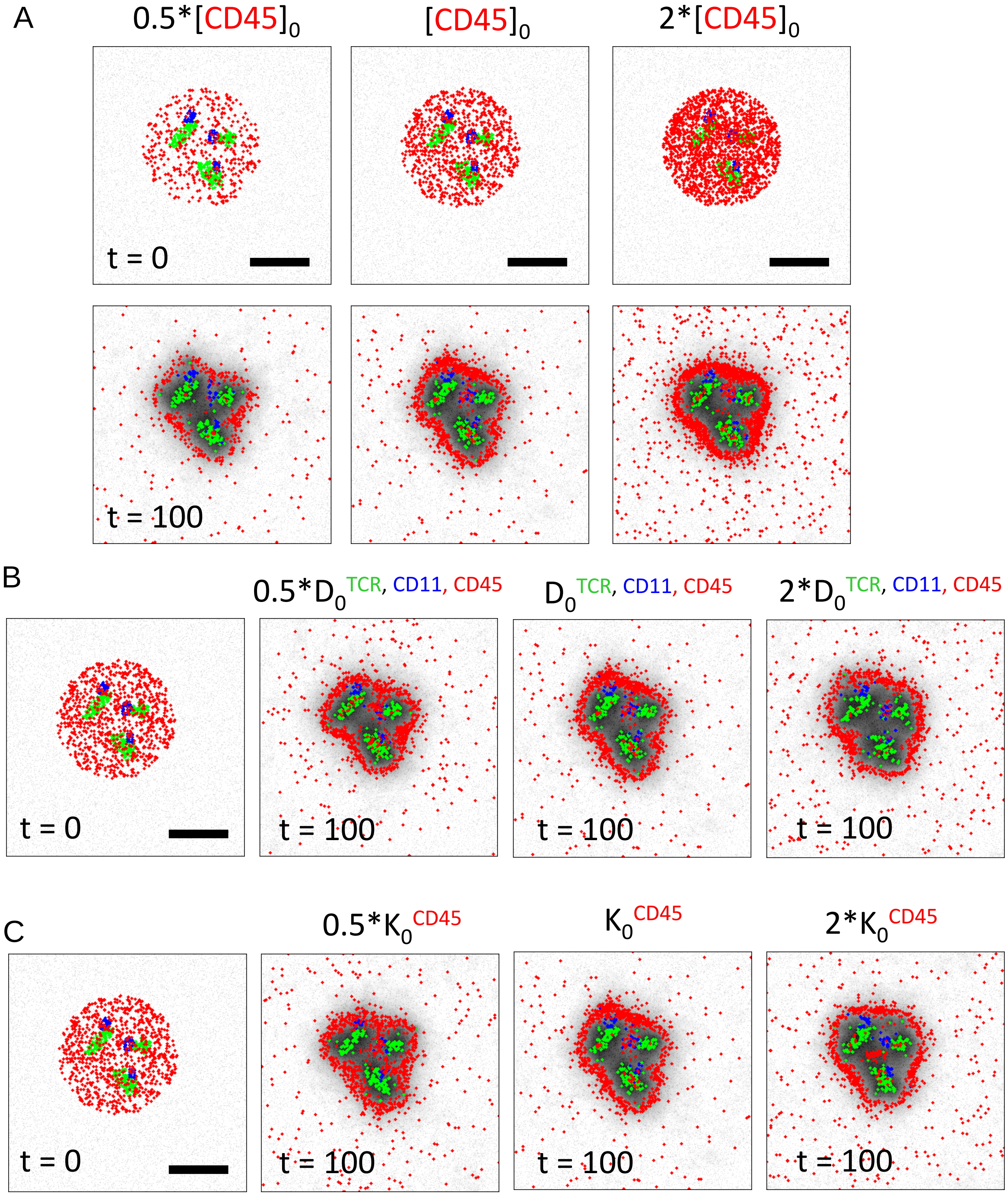
A
0.5*[CD45]$_0$
[CD45]$_0$
2*[CD45]$_0$
t = 0
t = 100
B
0.5*D$_0$$^{TCR, CD11, CD45}$
D$_0$$^{TCR, CD11, CD45}$
2*D$_0$$^{TCR, CD11, CD45}$
t = 0
t = 100
t = 100
t = 100
C
0.5*K$_0$^{CD45}
K$_0$^{CD45}
2*K$_0$^{CD45}
t = 0
t = 100
t = 100
t = 100

Figure S3

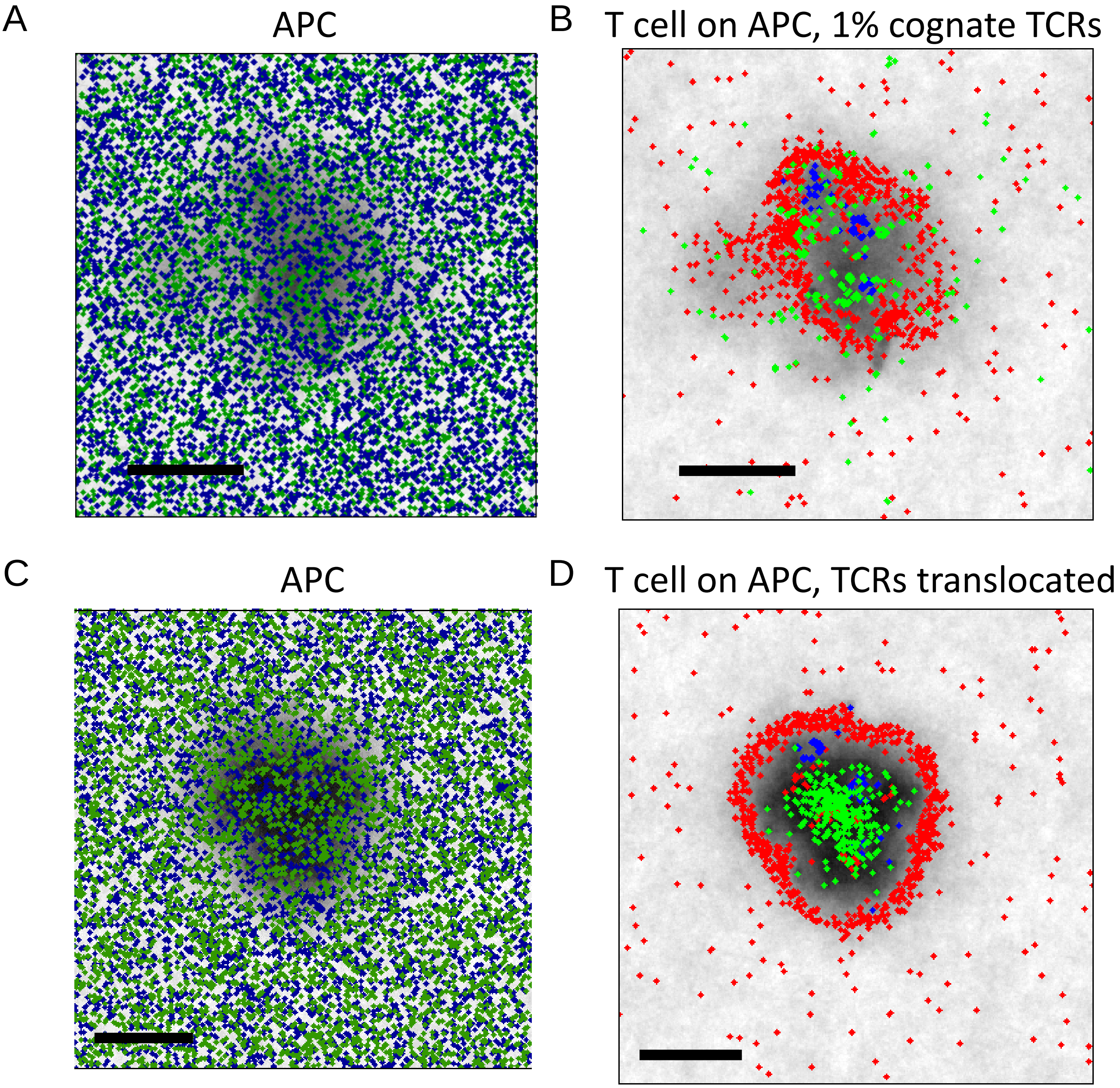
A
APC
B
T cell on APC, 1% cognate TCRs
C
APC
D
T cell on APC, TCRs translocated
