# Supplementary material for: InterCells: A Generic Monte-Carlo Simulation of Intercellular Interfaces Captures Nanoscale Patterning at the Immune Synapse
Source: Front Immunol. 2018 Sep 11;9:2051. doi: 10.3389/fimmu.2018.02051 (PMC6141710; doi:10.3389/fimmu.2018.02051)
Supplement: Supplementary file 4 [file Data_Sheet_1.pdf]

InterCells User's Guide (version 1.2)

Table of Content:

**1. About**	**1**
**2. The structure of the simulation**	**1**
**3. Operating the simulation**	**3**
**4. An example – Self-clustering of membrane proteins**	**20**
**5. References**	**23**

1. About

1.1 General

InterCells is an agent-based Monte-Carlo simulation of user-defined cellular interfaces. The simulation allows for membrane molecules, embedded at intercellular contacts, to diffuse and interact, while capturing the topography and energetics of the plasma membranes of the interface. The simulation was developed following Weikel and Lipowsky[1]. The code is written in Matlab (MathWorks). The simulation is open-source, interactive and modular.

The goal of the simulation is to facilitate an accessible, rapid, yet quantitatively critical feedback for generating experimentally testable hypotheses and the adaptation of working models in an iterative way.

1.2 Requirements

The simulation runs on MATLAB 2012a or newer versions. It runs on a standard PC. Typical runtime of the simulations takes ~5min for 10,000 iterations on a PC with i7 quad processor. Such simulation include two interacting surfaces, each of 400x400 pixels.

1.3 Installing the simulation

All simulation files are provided are available online on Github (https://github.com/ShermanLab/InterCells). These files should be downloaded to the User's computer under a directory that can be accessed by Matlab.

1.4 Licensing and citation

The simulation is freely available under the free GNU General Public License.

The User is requested to cite Neve-Oz et al. [2] in any publication in which the simulation was used.

2. The structure of the simulation

The simulation structure can be divided into multiple layers (Fig. 1). The first layer includes the input parameters and their respective GUI. The second layer includes the core of the simulation, where the physical models and the simulation algorithms are embedded and run. The third layer includes the output of the simulation. The fourth layer includes multiple analysis tools that are provided for quantitative interpretation of the simulation results and for their comparison with experimental data. A schematic description of the cell interface simulation is provided in Fig. 2 below.

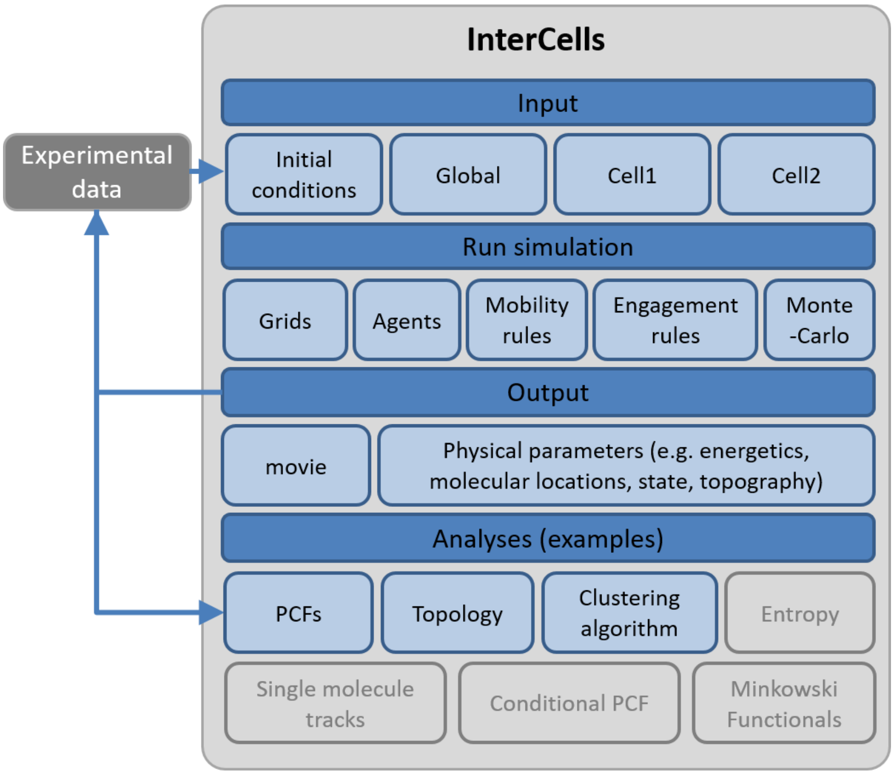

Fig 1. A schematic description of InterCells.

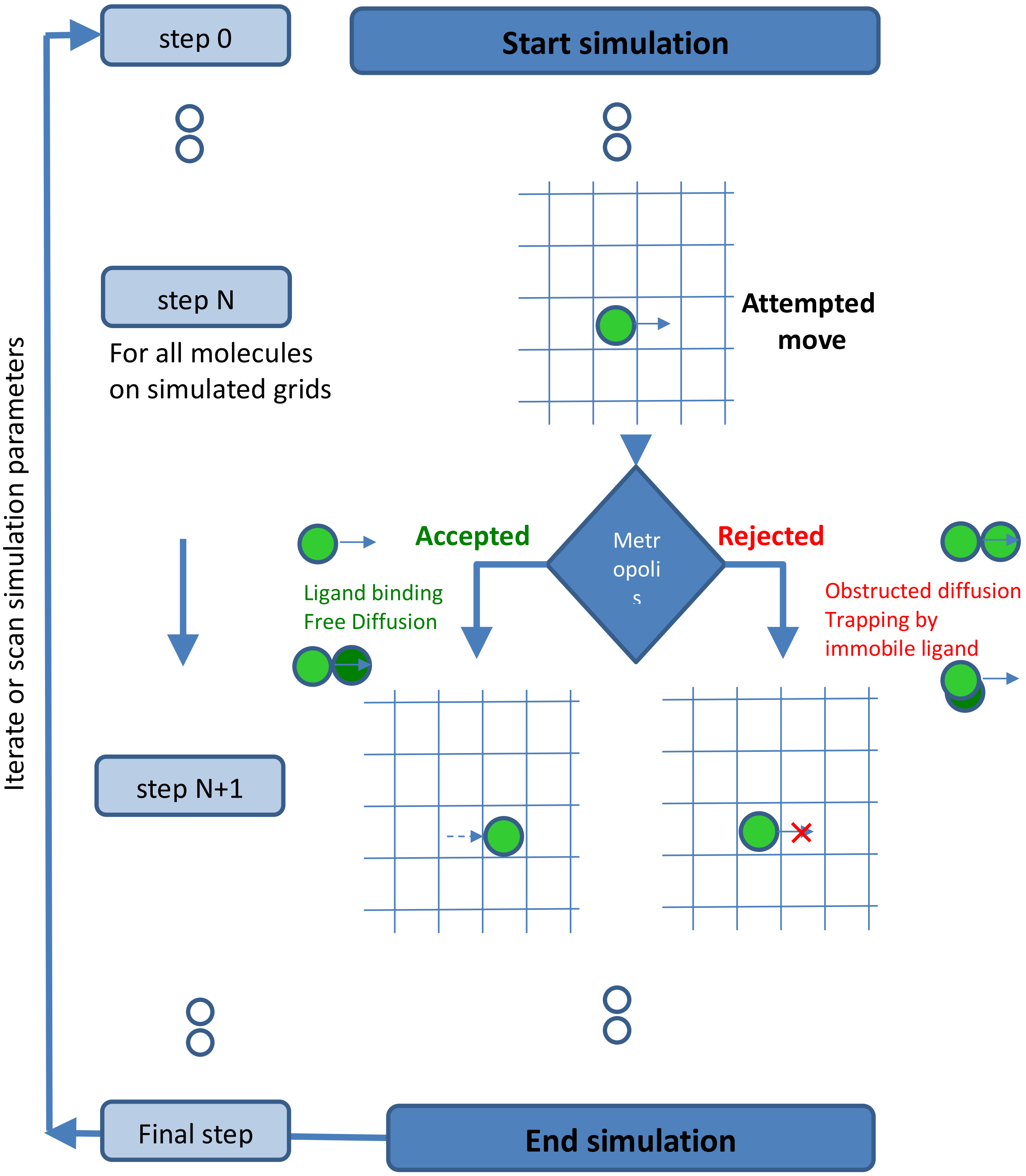

Fig 2. A schematic description of the cell interface simulation.

3. Operating the simulation

3.1 Loading and running the simulation

For loading the simulation, the User should type 'start_interface_simulation' and press 'Enter' in the Matlab command line. After running this command, the main window of the simulation GUI appears (Fig. 3). All inputs and outputs of the simulation can be accessed and changed through this main window (Fig. 3). After changing the desired parameters, the simulation can start by pressing the 'Run' button in the window.

3.2 Input

The code uses default parameters for its initial set-up, as described below. The user can modify all simulation parameters through the GUI, as graphically instructed in Figs 3-7 before running the simulation.

3.3 Output

Quantifiable readouts of the numeric simulations include the position and state of individual proteins, the morphology of the PM and their energetics. Visualization tools are provided for showing the simulation results. For instance, live evolution of molecular patterning is provided during the simulation run. The patterns can then be shown for each step individually, or as a movie. All parameters of the simulation are automatically saved into a 'Results' folder.

Examples of simulation outputs for the concrete example of the IS are provided in Neve-Oz, et al [2].

3.4 Analyses

InterCells integrates multiple statistical tools for quantitative analyses and interpretation of the results. Our tools include clustering algorithms and second-order statistics [3,4], and the topology analysis (Fig. 4). These tools are important for the quantitative comparison between results from experiments and from simulations.

3.5 Initial settings of simulation parameters

Simulation parameters are divided into global parameters (Table 1), parameters of the plasma membrane of two interacting cells or of two interacting interfaces (Table 2), molecules parameters (Table 3), and analyses parameters.

An initial simulation data is formed after the setup starts. The initial setting is based on default parameters that define the behavior of all the entities in the simulation.

The simulation parameters are drawn from a specific simulation of pattern formation in the immune synapse (SI) between T cells and antigen presenting cells (APCs), as described in detail in Neve-Oz, et al [2]. This manuscript should be cited in reference to the simulation.

Below, we provide considerations for parameter choice in our simulated example. The user may use these parameters (again, specified as defaults) or may change any of these parameters, as explained in the following section (3.6).

3.6 Global parameters (Table 1)

- The typical grid size (400x400 pixels) and a pixel size of $10x10nm^2$ were chosen to include a region of interest (ROI) within a cell footprint, with a typical area of $10x10\mu m^2$. Considering a ROI size of $2x2\mu m^2$ would leave a wide enough margin (e.g. ~50-100pixels), such that boundary effects are minimized. A bigger grid size minimizes the effect of the boundary, yet requires longer (actual) simulation time, computational power and memory.
- The simulation time should have a fast enough resolution to capture molecular pattering due to molecular motion via diffusion or transport. However, similar considerations that limit the realistic grid size of the simulation (discussed above), may restrict the iteration time, overall simulation time, the save rate and the overall number of runs (Table S1).
- 'Dynamics' parameters determine the number of steps for the Metropolis criterion. For instance, a two-step Metropolis requires first an unbinding energy to be obtained and then a rebinding or escape to take place. Stick time allows for non-specific adhesion to restrict the unbinding of molecules over the user-defined time.
- Experimental systems (being either artificial mimics of interacting cells) may contain non-specific adhesive interactions. Thus, we provide an option for the user to set such non-specific interactions via the commonly used reagent poly-L-lysine (PLL). Of course, this feature could represent any other type of non-specific, uniformly distributed interactions.
- The number of runs allows for the user to capture the statistical variations in the simulation outcomes. This is also useful for quantifying the statistical significance of altering simulation parameters.

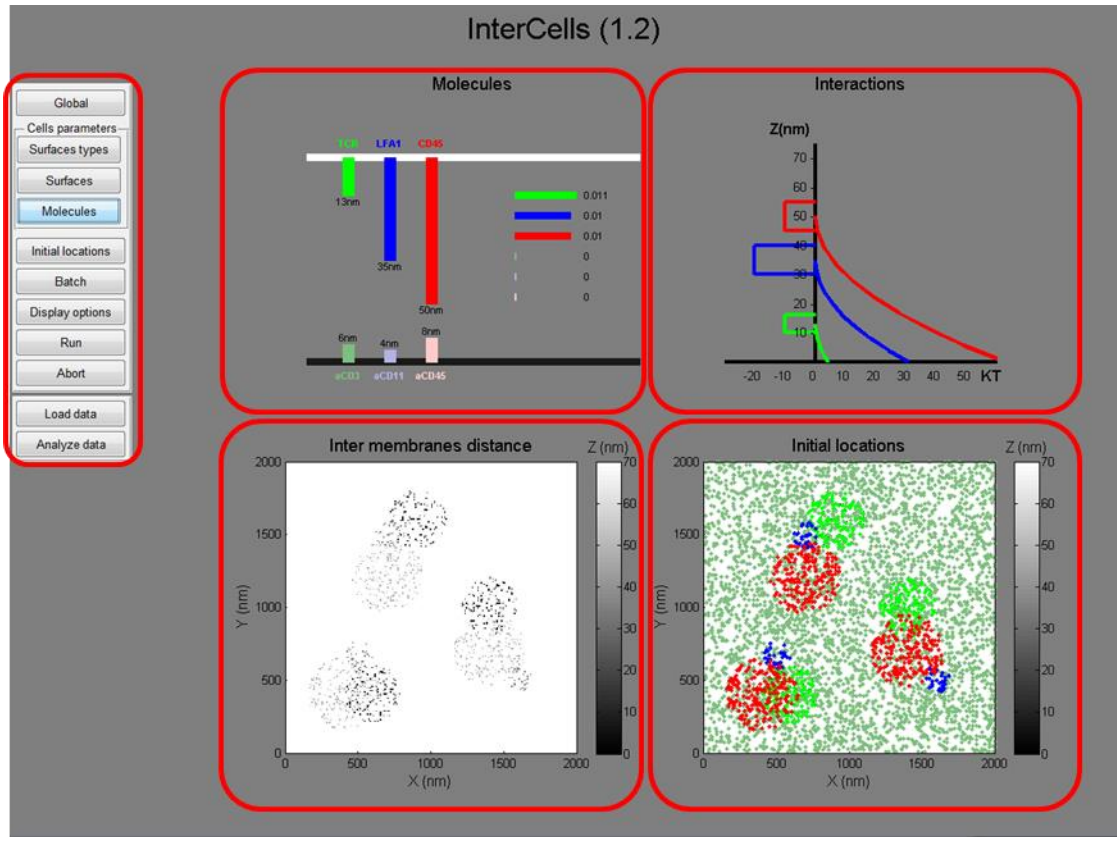

Fig 3. The main window of the GUI. The panel shows the functional parameters of the simulation. The GUI is controlled by the interactive menu at the left (red enclosure).

Table 1. Default Global parameters

Default global parameters		
Array		Units
size x	400	pixels
size y	400	pixels
Pixel size	10	nm
Times		
Iteration time	0.01	sec
Simulation time	100	sec
Experiment frame rate	2.5	sec
Save rate	100	iterations
Dynamics		
Metropolis steps	2	1,2
Stick time	100	sec
Poly-L-lysine		
Binding strength	-4	KT
Use poly-L-lysine	Yes	Yes / No
Number of runs		
Number of runs	1	
Membrane domains		
Use adhesive domains	Yes	Yes/No
Adhesion strength	-4	KT
Circular domain radius	20	nm
Actin		
Use actin	No	Yes/No
Actin rigidity	100	KT

3.7 Membrane parameters (Table 2)

The membrane parameters define the overall physical characteristics of the simulated interfaces. Such interfaces may describe specific cell types (here, T cells and APCs) or they can describe artificial mimics of these cells, such as coverslips or glass-supported lipid bilayers. Specific parameters are taken from the literature, as specified in right-most column of Table 2.

- The rigidity of the surface may include its global minimum or maximum rigidity (in units of KT). It will also include a future option to define local variations (via a dedicated interactive tool) in the rigidity, such as by the presence of cortical actin.
- The diffusivity of the molecules – an effective decrease in molecular diffusivity due to membrane viscosity
- The initial height Z of the surface (in nm) and its global restrictions.
- The membrane fluctuations are assumed to have a sigma value dz of 1nm

Table 2. Default Membranes' parameters

**Default membranes' parameters**						
	T-cell	APC	Coverslip	Lipid bilayer	Units	Refs
Rigidity						
Rigidity	25	25	10^6	10^6	KT	[26]
Minimum rigidity	25	25	10^6	10^6	KT	[26]
Maximum rigidity	100	25	10^6	10^6	KT	[26]
Local rigidity	No	No	10^6	10^6	Yes / No	
Diffusivity						
Diffusivity	1	1	0	1	[0 1]	
Minimum diffusivity	1	1	0	1	0	
Maximum diffusivity	1	1	0	1	1	
Local diffusivity	No	No	No	No	Yes / No	
Height						
Z_0	70	0	0	0	nm	
Minimum height	10	0	0	0	nm	
Maximum height	100	0	0	0	nm	
Sigma dz	1	1	0	0	nm	[26]

3.8 Membrane parameters (Table 3)

Multiple parameters describe the individual properties of each molecular type, participating in the simulation.

- Names and colors are useful for the graphical representation of the molecules.
- Vertical molecular sizes are drawn from the literature. Their footprint was set to 1 pixel (i.e. occupying $10x10nm^2$). Importantly, our simulations restricts the occupancy of each pixel in one grid to one molecule. Thus, no two molecules (regardless of their type) may occupy the same pixel on the same grid. This restriction will likely be removed in future updates of the simulation.
- Interaction potentials – In the simulation, molecules may act as either repulsive springs, attracting agents with either ligand-specific or non-specific attraction, or a combination of these interactions. The interaction potentials are graphically described and updated in the GUI.
- The diffusion of each molecule may be defined. Values are taken from published measurements.
- Molecules may also demonstrate the ability for transport across the cell footprint. Such transport has been found for multiple signaling molecules, including TCRs, ZAP-70 and SLP-76 in mature immune synapses. Molecular transport may be conducted by motors along cytoskeletal segments.

- Molecules may also demonstrate tendency for self-clustering. In our simulations, we often considered such tendency for TCR and CD11 (LFA-1) which are often found in clusters. We provide below a second example of using the simulation to study the effect of this property and of molecular trapping on its clustering at the PM.

Table 3. Default Molecules' parameters

Default molecules' parameters												
	T-cell			Coverslip			APC		Lipid bilayer		Units	Refs
Name/Colour	TCR	LFA-1	CD45	aCD3	aCD11	aCD45	pMHC	ICAM	pMHC	ICAM		
Type number	1	2	3	1	2	3	1	2	1	2		
sizes												
Vertical size	13	35	50	0	0	0	0	0	0	0	nm	[26]
Lateral size	10	10	10	10	10	10	10	10	10	10	nm	[26]
Area	1	1	1	1	1	1	1	1	1	1	pixels	[26]
potentials												
Potential width	6	10	10	0	0	0	0	0	0	0	nm	[26]
Binding bottom	10	30	45	0	0	0	0	0	0	0	nm	[26]
Binding top	16	40	55	0	0	0	0	0	0	0	nm	[26]
Binding strength***	-10	-20	-10	-10	-10	-10	-10	-20	-10	-10	KT	[26]
k spring**	0.1	0.1	0.1	0	0	0	0	0	0	0	KT/nm^2	[26]
Force membrane to molecule height	Yes	Yes	No	No	No	No	No	No	No	No	Yes/No	
Diffusion and distributions												
Diffusion constant**	0.01	0.01	0.01	0	0	0	0.01	0.01	0.01	0.01	µm^2/sec	[26]
Global density**	300	300	300	300	300	300	300	300	300	300	#/µm^2	[18]
Cluster density*	1000	1000	1000	1000	1000	1000	1000	1000	1000	1000	#/µm^2	[19]
Density of clusters*	0.8	0.8	0.8	0.8	0.8	0.8	0.8	0.8	0.8	0.8	#/µm^2	
Dynamics												
Self-clustering	Yes	Yes	No	No	No	No	No	No	No	No	Yes/No	
Self-clustering binding range	10	10	10	0	0	0	10	10	10	10	nm	
Self-clustering P_{on}	0.995	0.995	0	0	0	0	0	0	0	0	[0 1]	
Self-clustering P_{off}	0.005	0.005	1	1	1	1	1	1	1	1	[0 1]	
Transport												
Use transport	Yes	No	No	No	No	No	No	No	No	No	Yes/No	
Transport speed	19	0	0	0	0	0	0	0	0	0	nm/sec	[29]

Comments:

* - sensitivity analysis is provided in Fig. S1
**- sensitivity analysis is provided in Fig. S2
*** - sensitivity analysis is provided in Fig. S3

1.1 Modifying the simulation parameters and initial conditions

We provide below a graphical explanation on how to modify the simulation parameters before its run (Figs. 4-11).

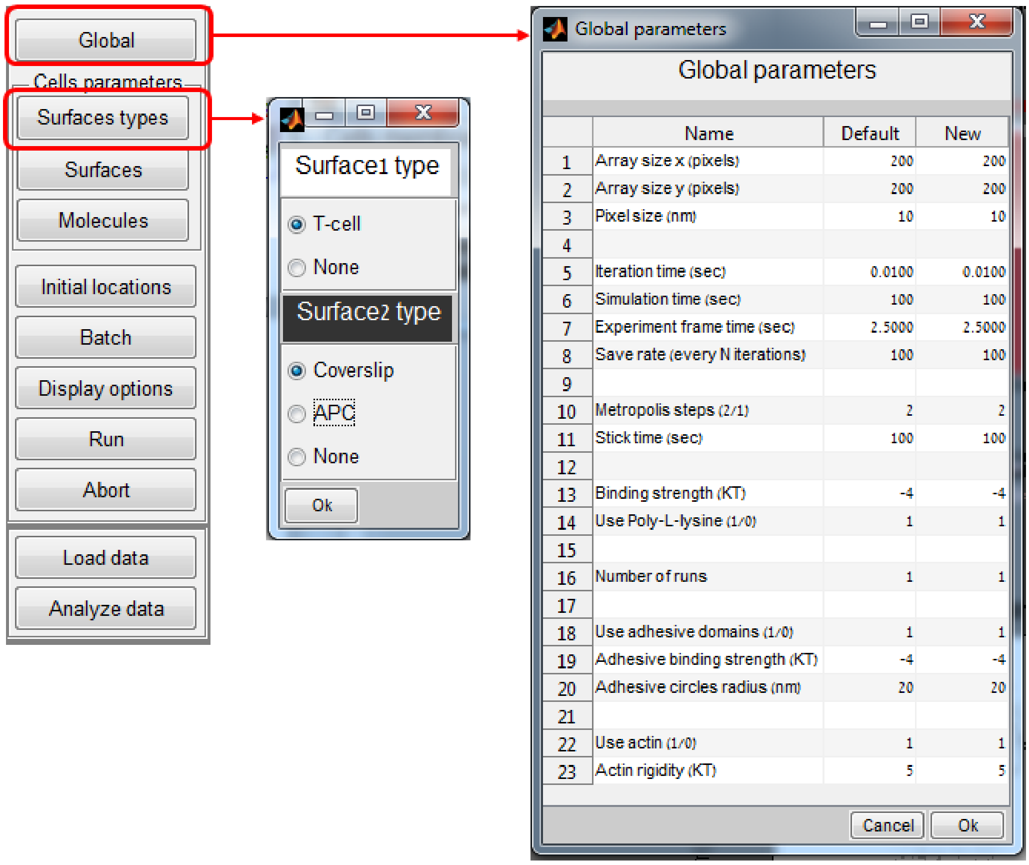

Fig 4. Setting the global parameters of the simulation

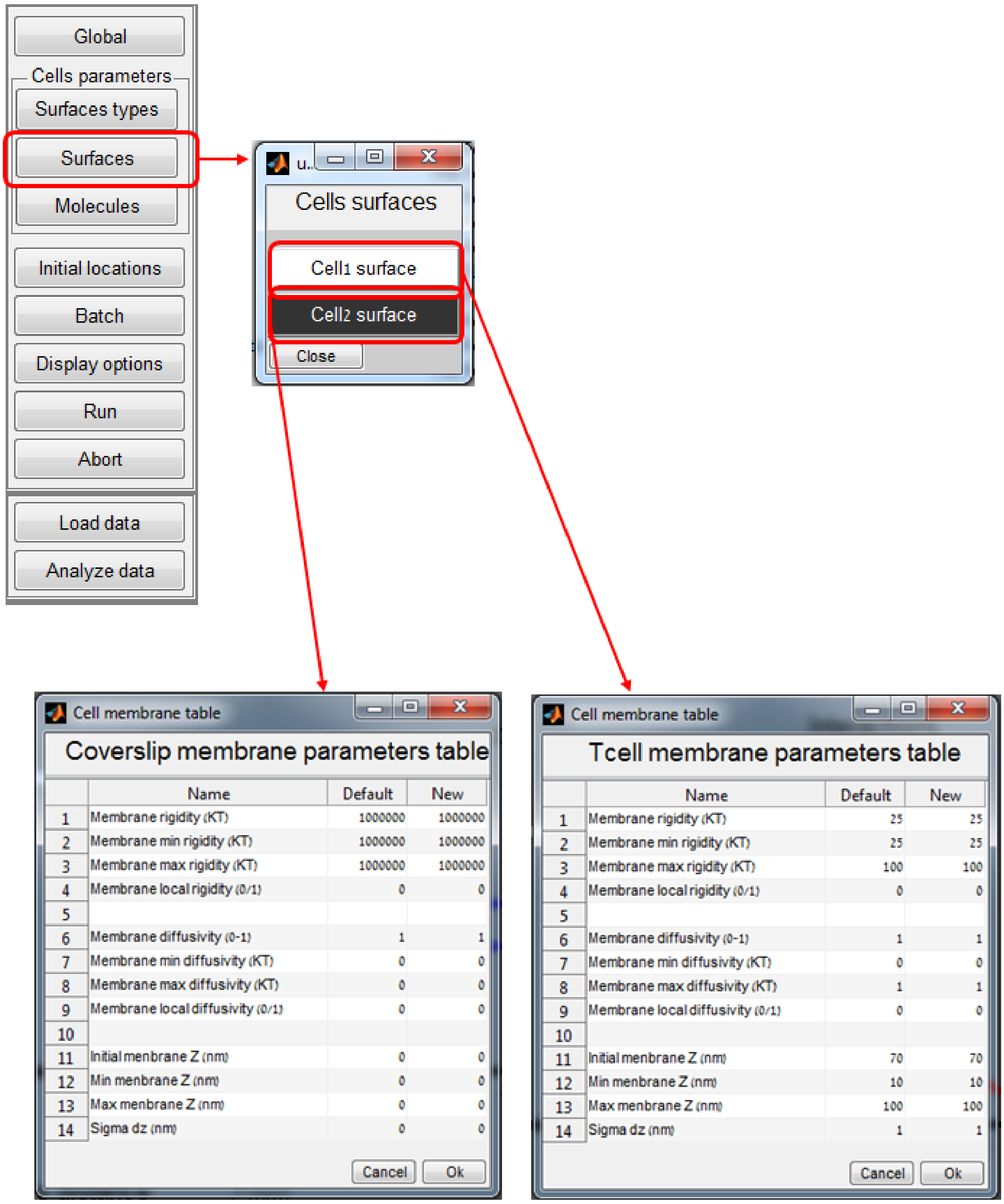

Fig 5. Setting the Membranes' parameters

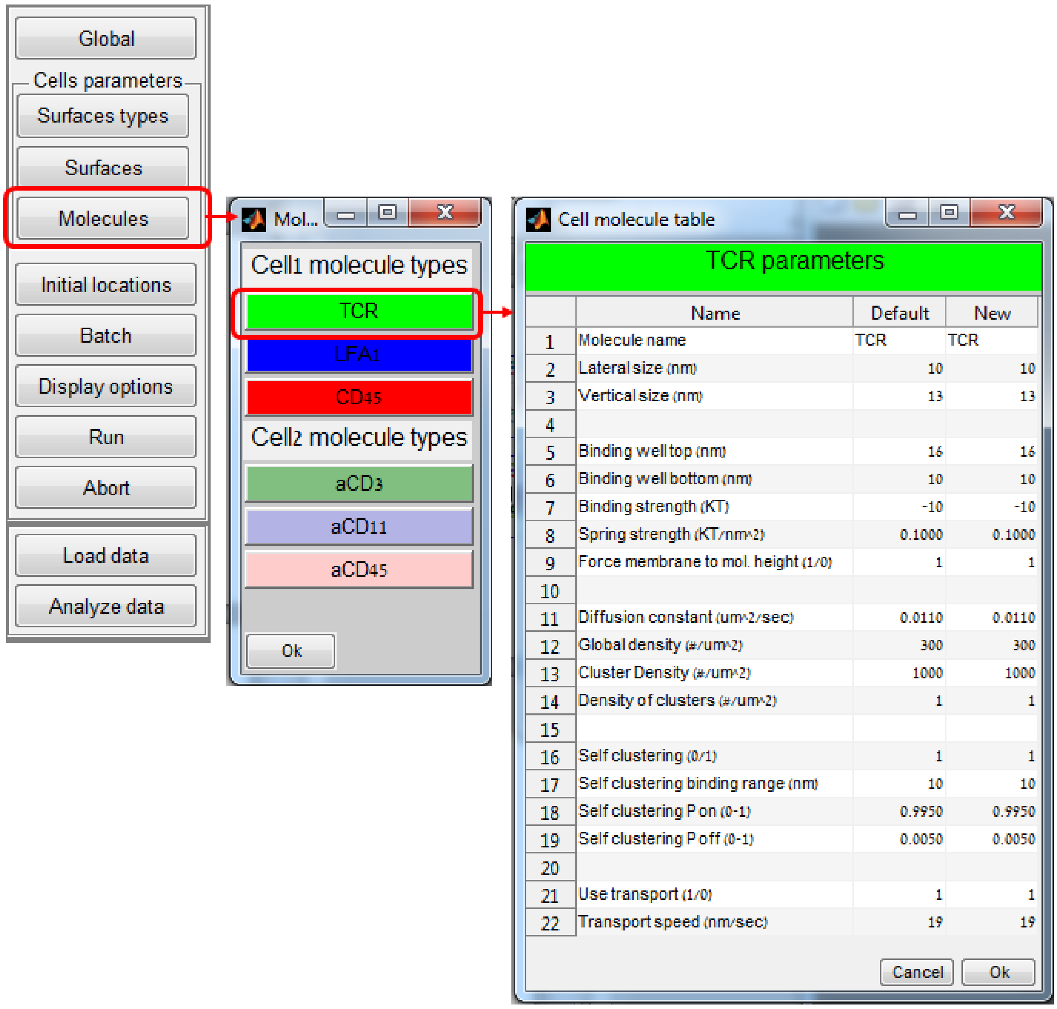

Fig 6. Setting the Molecules' parameters

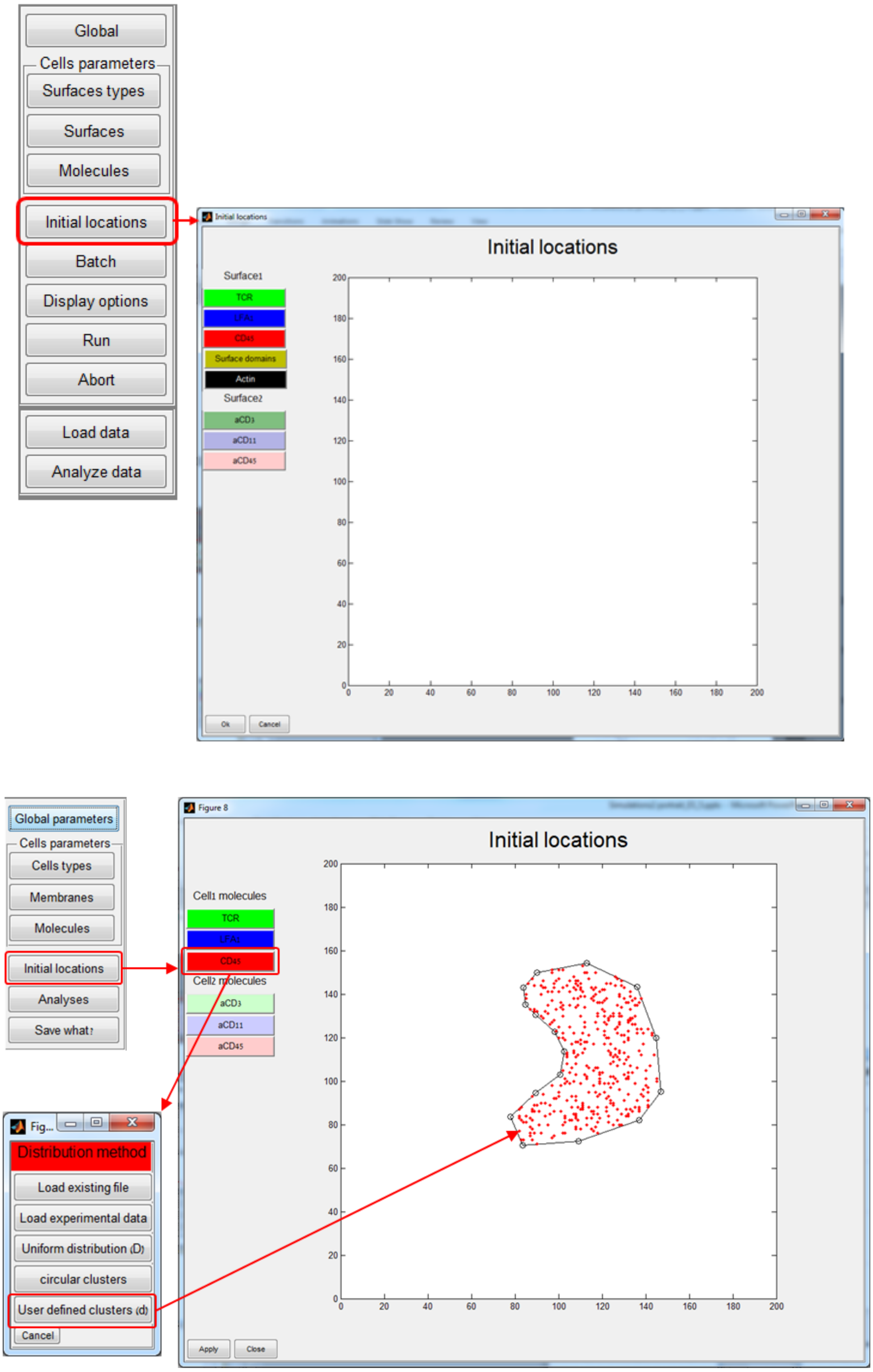

Fig 7. Setting the initial placement of molecules. The placement of each molecule type can be spatially defined in the simulation. For instance, a cluster can be defined by the user via the computer Mouse. Left Mouse clicks in the window set the cluster edges. A single right click of the Mouse sets the last edge. The defined molecules are than distributed randomly within the cluster.

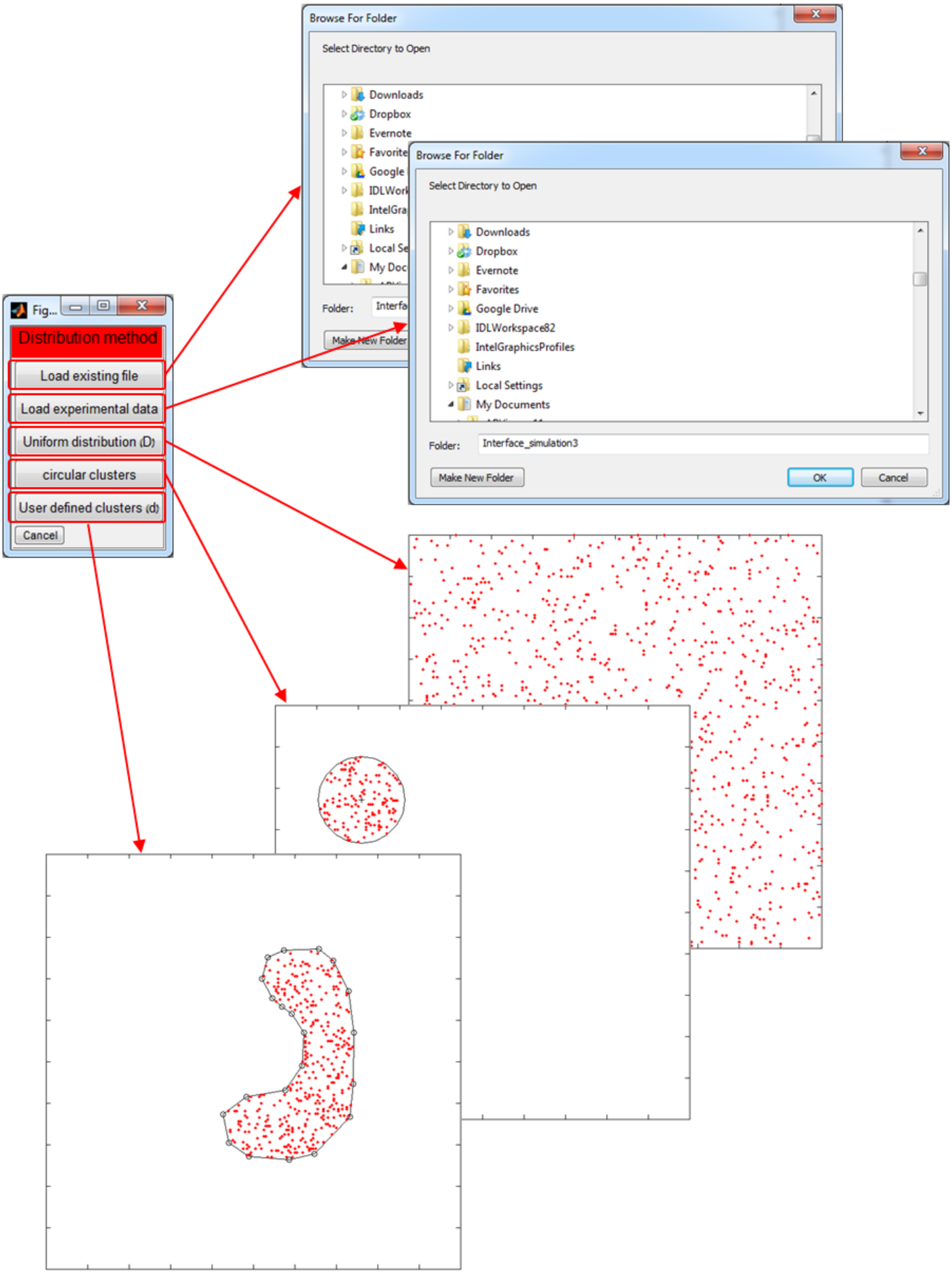

Fig 8. Setting the initial placement of molecules (continued). Alternative ways to distribute molecules include their uploading from experimental data. This way results in hybrid simulations. Likewise, molecular coordinates generated by any other means can be imported. Otherwise, similar to the instructions in Fig. 7, molecules can be distributed uniformly in circular clusters (rather than in user defined polygons). A uniform distribution of molecules across the simulated field is another useful option, by clicking the 'uniform distribution' button in the menu.

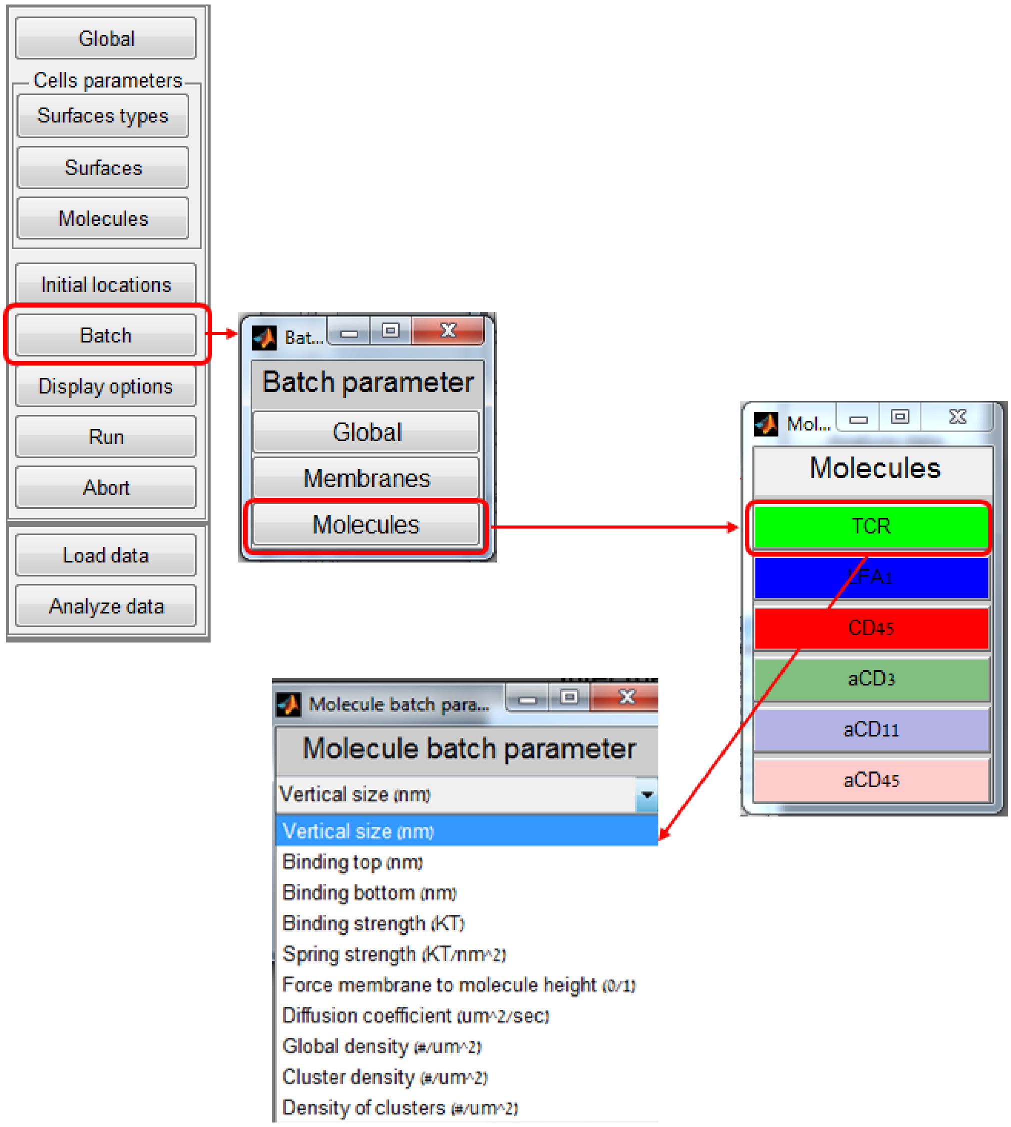

Fig 9. Setting parameters for batch runs

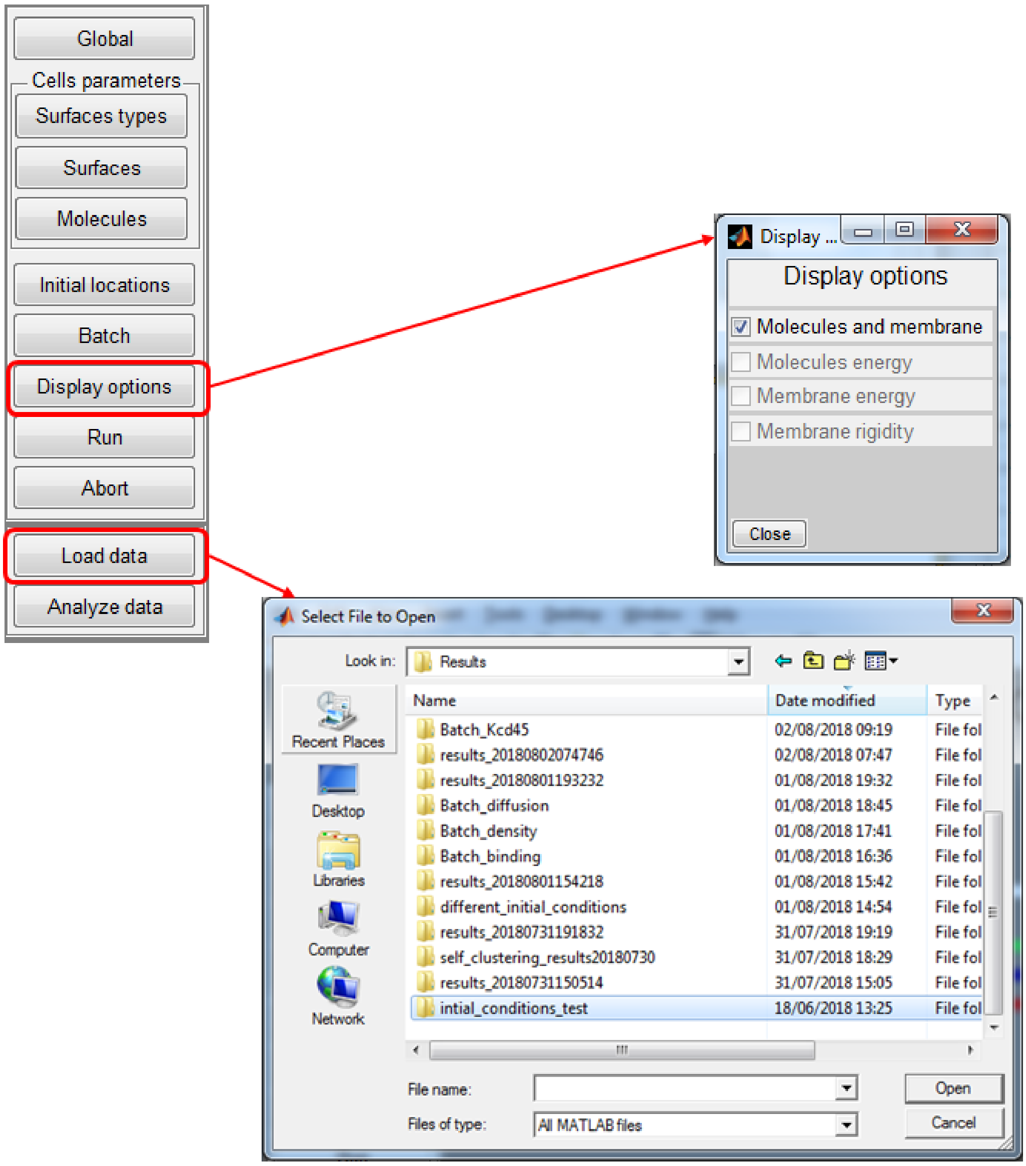

Fig 10. Loading data for analysis. Data can be either experimental or simulated.

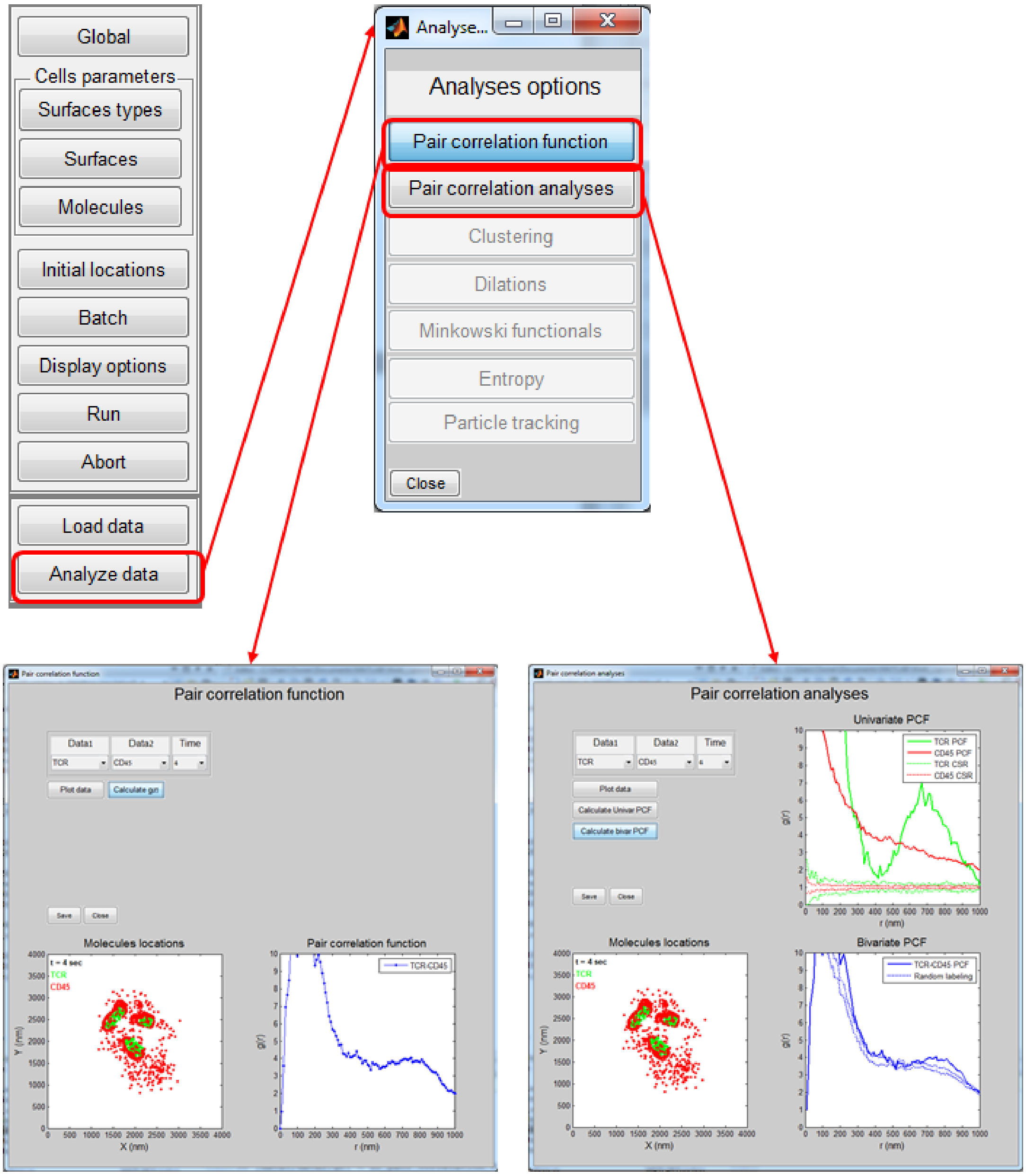

Fig 11. Data analyses. Univariate and bivariate pair correlation functions (PCFs) can be calculated for different molecular types and at chosen time points along the simulation. Null hypotheses of 'complete spatial randomness' (CSR) and 'Random labeling' can be automatically generated to evaluate the significance of the results.

4. An example – Self clustering of membrane proteins

4.1 Introduction

With the advancement of immunogold-labeling TEM and superresolution optical microscopy, multiple proteins have been shown to form nanoscale self-clusters at the plasma membrane of cells. Multiple such proteins are involved in T cell activation, including the TCR, LFA, LAT, and others[5,6]. However, molecular self-clustering likely occurs in all cells, and include GPI-anchored proteins, receptors such as GPCRs, Ras proteins, etc.

The mechanism of molecular self-clustering at the plasma membrane and its role in signaling is currently under intensive study. Mechanisms that promote self-clustering may include protein-protein interactions, protein-lipid interactions. Such interactions can be mediated by structured elements such as lipid rafts, adaptor proteins, protein confinement by cortical actin, molecular traps, and more. Function may include regulation of the protein (e.g. via endocytosis) and synergy in signaling via binding of effector proteins or cooperativity.

Here, our goal is to demonstrate how our simulation can serve to study mechanisms of protein self-clustering at the plasma membrane of a cell. The protein and the membrane are kept intentionally general. The proteins are given a property of self-affinity. We also embed certain static domains at the membrane that can trap the protein. We then let the proteins diffuse and interact and study the self-clustering of the proteins after 100s.

4.2 Simulations

We simulated 1200 proteins of one specific type, diffusing in a 2μmx2μm field with a diffusion coefficient of 0.011$\mu m^2/s$. Self-clustering was defined by a P_{on}=0.995 upon encounter and a P_{off}=0.005. Trapping domains had a circular shape with a diameter of 50nm. Binding energy of molecules to traps was -4$K_B T$. Simulations took 10K iterations (comparable to 100s).

4.3 Results

Self-clustering of the protein under study is quantified via the PCF statistics (under the Analyses). Our results are shown in Fig. 12 below. The proteins with no self-interaction and no trapping domains show no apparent clusters (Fig. 12A) and a flat PCF with a value of 1 (Fig. 12E, blue line), as expected for a Poisson process. Including trapping domains at the membrane results in small clusters that are localized at the traps and a large fraction of monomers (Fig. 12B). It also results in an increased PCF (Fig. 12E, red line). Self-clustering of the protein results in nanoclusters that are distributed randomly and have variable sizes (Fig. 12C). The

PCF for self-clustering shows further increase in the PCF curves (Fig. 12E, green line). Combining the mechanisms of trapping domains and self-clustering shows pronounced nanoclusters that include most of the proteins and are nucleated mainly at the traps. As expected, the PCF for the combination of the mechanisms shows the highest values at all length-scales (Fig. 12E). The height of the PCF curve indicates here the highest level of self-clustering compared to a Poisson process. The decay of the curve to a value of 1 indicates the generation of the largest clusters under these conditions.

4.4 Conclusions of the example

We conclude that our simulation can assist in the study of clustering mechanisms of proteins at the PM. The experimental access to individual clustering mechanisms is typically very hard, and often impossible. In contrast, such mechanisms can be studied seamlessly through simulations and yield predictive results for experiments, in return.

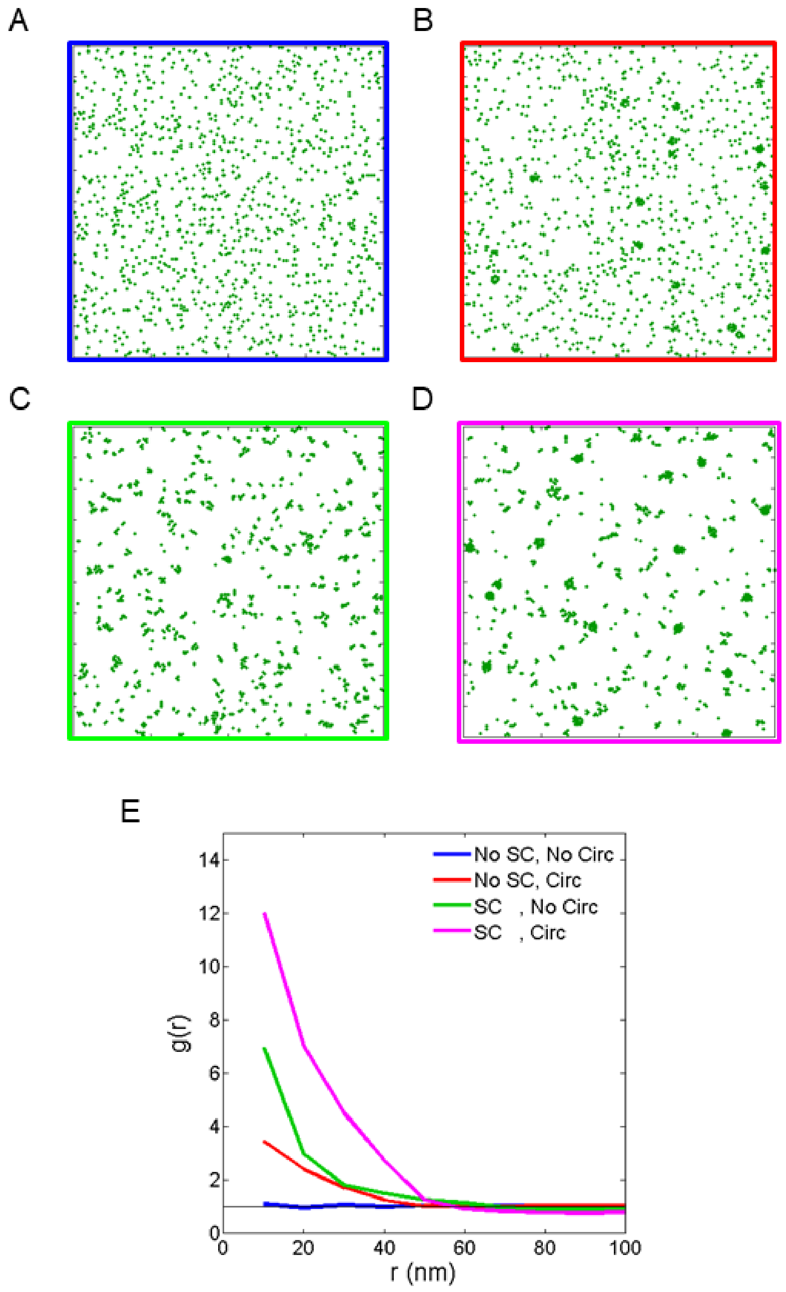

Fig 12. Molecular self-clustering at the plasma membrane. (A-D) Molecular organization of the molecules are shown after 10K iterations (100s), under the following conditions: (A) No trapping ('No Circ') and no self-clustering ('No SC'); (B) Trapping ('Circ'); (C) Self-clustering ('SC'); and (D) Trapping and self-clustering ('SC, Circ'). (E) The PCF statistics for the different simulation conditions of A-D.

2. References

1 Weikl, T. R. & Lipowsky, R. Pattern formation during T-cell adhesion. *Biophys J* **87**, 3665-3678, (2004).

2 Neve-Oz, Y. S., J. Razvag, Y., Sherman, E. InterCells: a generic Monte-Carlo simulation of intercellular interfaces captures nanoscale patterning at the immune synapse. *Submitted*, (2018).

3 Sherman, E., Barr, V., Manley, S., Patterson, G., Balagopalan, L., Akpan, I., Regan, C. K., Merrill, R. K., Sommers, C. L., Lippincott-Schwartz, J. & Samelson, L. E. Functional nanoscale organization of signaling molecules downstream of the T cell antigen receptor. *Immunity* **35**, 705-720, (2011).

4 Sherman, E., Barr, V. A. & Samelson, L. E. Resolving multi-molecular protein interactions by photoactivated localization microscopy. *Methods* **59**, 261-269, (2013).

5 Lillemeier, B. F., Pfeiffer, J. R., Surviladze, Z., Wilson, B. S. & Davis, M. M. Plasma membrane-associated proteins are clustered into islands attached to the cytoskeleton. *Proc Natl Acad Sci U S A* **103**, 18992-18997, (2006).

6 Sherman, E., Barr, V. & Samelson, L. E. Super-resolution characterization of TCR-dependent signaling clusters. *Immunol Rev* **251**, 21-35, (2013).
